# The evolutionary basis of elevated testosterone in women with polycystic ovary syndrome: an overview of systematic reviews of the evidence

**DOI:** 10.3389/frph.2024.1475132

**Published:** 2024-09-30

**Authors:** Aiden Bushell, Bernard J. Crespi

**Affiliations:** Department of Biological Sciences, Simon Fraser University, Burnaby, BC, Canada

**Keywords:** polycystic ovary syndrome, evolution, testosterone, strength, muscularity, dominance

## Abstract

Polycystic ovary syndrome (PCOS) exhibits high prevalence and heritability despite causing negative impacts on fertility and fecundity. Previous hypotheses have postulated that some PCOS-associated traits, especially above-average levels of testosterone, were associated with benefits in ancestral environments. As such, PCOS would represent, in part, a maladaptive extreme of adaptations related to relatively high testosterone. To evaluate this hypothesis, we conducted a series of systematic literature reviews on the associations of testosterone levels, and prenatal testosterone metrics, with measures of strength, robustness, muscularity, and athleticism in females. We also systematically reviewed the literature on associations of testosterone with dominance in females and reviewed archaeological evidence concerning female strength and muscularity and its correlates. The main findings were fivefold: (1) elevated testosterone levels were generally associated with higher strength, muscularity and athleticism in females; (2) females with PCOS showed notable evidence of increased strength, muscularity, and athleticism compared to controls; (3) females with higher testosterone levels exhibited clear evidence of high dominance, (4) despite evidence that higher testosterone is linked with higher bone mineral density in healthy females, PCOS was not clearly associated with this phenotype; and (5) archaeological evidence from osteology, and data from some current small-scale societies, indicated that females often exhibit substantial levels of muscularity. Overall, the hypothesis that relatively high levels of testosterone are associated with benefits to females in some contexts was largely supported. These results provide evidence for the “maladaptive extremes of adaptation” model, with implications for treatment of females with PCOS and for future research.

## Introduction

Some female-limited diseases with negative impacts on fertility and fecundity are present at high frequencies within contemporary populations. For example, polycystic ovary syndrome (PCOS) and endometriosis each demonstrate a prevalence of about 5%–15% (1–3) and a heritability of 50%–70% (4–6), while notably reducing lifetime fitness (7, 8). How and why have risks for such diseases evolved, and how are they maintained?

Hypotheses for the maintenance of common, deleterious diseases can be addressed by several evolutionary factors including mismatches, trade-offs, and extremes of adaptations (9–11). In each case, maladaptive aspects of the disease reflect adaptations, and their constituent trade-offs, that have become dysregulated in particular ways. For example, risks and symptoms of endometriosis show evidence of involving evolutionary trade-offs and extremes of adaptations related to female life histories and sexual selection (10–13). Low testosterone levels, earlier menarche, and earlier menopause are all correlates of endometriosis that are consistent with a fast life history, with increased investment in earlier reproduction (13). For any given reproductive disorder, the challenge is to determine how the risks and symptoms are related to adaptations and trade-offs, in the contexts of current and ancestral environments.

In this paper, we evaluate the primary evolutionary hypothesis described thus far for the maintenance of PCOS, one of the most common disorders of female reproduction. We describe the symptoms and diagnostic criteria for PCOS, and then briefly explain the main hypothesis described thus far for explaining the evolutionary basis of risk for PCOS: that non-clinical PCOS-associated traits, especially relatively high levels of testosterone and associated dominance, strength, robustness, and muscularity, are contextually advantageous especially in the context of higher survival (9, 11, 14–18). Some evidence relevant to this hypothesis has been described previously, but data salient to testing the hypothesis has yet to be evaluated comprehensively. We do so here, using a series of systematic reviews that target key predictions of the hypothesis.

### Symptoms and diagnosis of PCOS

PCOS is characterized by some combination of three main criteria: (i) hyperandrogenism (high androgen production by the ovaries and the adrenal glands); (ii) anovulation or oligo-ovulation (absent or infrequent ovulation); and (iii) polycystic ovaries, which contain multiple small follicles, with curtailed development, that resemble cysts (9, 19). Hyperandrogenism in females with PCOS is also associated with the development of hirsutism, alopecia, and other health-related issues including insulin resistance, abdominal obesity, and pancreatic β-cell dysfunction [e.g., (20–24)]. High levels of insulin, found in about 65%–70% of women with PCOS, can increase the production of ovarian testosterone (23, 25). However, the presence of insulin resistance is not a diagnostic criterion for PCOS, and the relationship between this phenotype, PCOS, and high testosterone remains unclear [e.g., (26)]. The expression of high testosterone levels, low ovulation frequencies, and multifollicular ovaries grades continuously from clinical levels in females with PCOS to low levels, or absence of the traits, in healthy females (27).

High prenatal testosterone levels represent an important risk factor for PCOS, as indicated by experimental animal models (28–30) and by data linking PCOS risk with relatively long anogenital distances (AGD) and low second-to-fourth digit ratios (2D:4D) in humans (31–33). PCOS risk is also transgenerational, such that daughters of women with PCOS are about five times more likely than controls to develop the syndrome, in part as a consequence of exposure, *in utero*, to the high androgen levels of their mother (34). The links of prenatal testosterone levels with the symptoms and causes of PCOS indicate that a significant contributing factor to this disease is high prenatal testosterone levels, that program the developing hypothalamic-pituitary-gonadal (HPG) axis (35).

### Evolutionary causes of risk for PCOS

The main hypothesis proposed to explain the evolutionary basis for the risk and symptoms of PCOS, and its high frequencies in current populations, postulates that PCOS-associated traits, especially relatively-high testosterone, may have provided survival advantages to females in ancestral environments through several main effects: (1) increased strength, muscularity and dominance; (2) higher bone mineral density, increasing robustness and reducing fracture risk, especially in the context of heavy labor; and (3) higher body mass index through relatively-enhanced visceral fat storage, and higher propensity to develop insulin resistance, which may provide advantages under reduced-food conditions and when subject to infection (9, 11, 16–18, 36). In modern environments, increased food intake and reduced physical activity would result in maladaptation due to the effects of chronic obesity in increasing insulin resistance and testosterone levels, which interfere with ovulation and thus reduce fertility (15, 37, 38). As such, the evolutionary causes of PCOS risk include maladaptive extremes of testosterone levels, due predominantly to mismatches of current with ancestral environments, and dysregulation of trade-offs between investment in survival and reproduction.

### PCOS and the putative benefits of high testosterone in females

The main hypothesis addressed here for the maintenance and symptoms of PCOS is based primarily on the benefits of non-clinical PCOS-associated traits, most of which derive from relatively high levels of testosterone and its effects. By this hypothesis, females with relatively high testosterone exhibit advantages, especially in terms of strength, athleticism, dominance, and robustness, that in some social and ecological circumstances, can outweigh its evolutionary drawbacks in the context of reduced or delayed fertility. The hypothesis presented here does not view PCOS itself to be adaptive. Instead, it considers PCOS as a maladaptive extreme of processes and traits that can be adaptive in some circumstances.

In this article, we focus specifically on the hypothesis of benefits to relatively high testosterone in females that are related to strength, muscularity, robustness, and dominance. The sets of tests performed can be conceptualized into three sub-hypotheses. By the first hypothesis, healthy females with higher testosterone exhibit greater physical strength and athleticism. This hypothesis predicts that: (1) higher prenatal testosterone is associated with increased strength, muscularity, dominance, athleticism, and BMD in females, (2) elevated testosterone levels are associated with higher strength, muscularity, athleticism, BMD, and social and physical dominance in females, and (3) current testosterone levels in women with PCOS should more closely align with testosterone levels in female athletes than controls.

By the second hypothesis, females with PCOS should exhibit greater physical strength and athleticism. This hypothesis predicts that: (1) females with PCOS show evidence of increased strength, muscularity, athleticism, and bone mineral density, (2) lean females with PCOS, an important subgroup that may be especially relevant to ancestral conditions and less affected by negative metabolic conditions, also exhibit higher strength, muscularity, and athleticism compared to controls, and (3) females with PCOS show higher levels of social and physical dominance than controls.

By the third hypothesis, females from ancestral environments and modern-small scale societies are expected to show evidence of greater strength and muscularity than females in modern Westernized societies. This hypothesis predicts that: (1) female specimens seen in the archaeological record exhibit evidence of increased bone strength and muscularity, where variation in these factors overlaps with relevant traits among males, and (2) females in some ancestral environments and modern small-scale societies exhibit evidence of substantial strength, muscularity, and athleticism, in the context of heavy labour or other activities associated with their local socioecology. These hypotheses were evaluated with a series of systematic reviews.

## Methods

A series of systematic literature reviews was conducted to test the predictions of each hypothesis. Details regarding the review methodology, including the search terms, databases searched, inclusion criteria and exclusion criteria, are provided in Table 1. Additional methods, such as calculations of percent differences, are provided in the footers of relevant tables. Given that the reviews are systematic, they are unbiased regarding study inclusion. Throughout the exposition, the term “female” refers to biological sex, in the context of XX chromosome complement.

**Table 1:** Methods for systematic literature reviews in relevant tables and supplementary tables.

	Search terms for Web of Science and PubMed	Web of Science search	PubMed search	Google Scholar search terms	Inclusion Criteria	Exclusion Criteria
Table 2	“Testosterone” and “athlete*” (using the AND term) with the results of “strength” “muscularity” “bone mineral density” “athleticism” (using OR terms), while excluding the results of “male” “men” “man” “boy” terms (using NOT terms)	March 30, 2023, returned 250 records	August 19, 2024, returned 181 records	“Testosterone” AND “female” AND “athlete” AND “strength” OR “muscularity” OR “bone mineral density” OR “athleticism”	*P*-values, correlations between serum or salivary T and measures of either strength, muscularity, BMD, or athleticism	Review articles, studies using only unhealthy populations (e.g., PCOS), combined sex analyses only, studies of males only, and transexual men or women only
Table 3	Testosterone” and “athlete*” (using the AND term) while excluding the results of “male” “men” “man” “boy” terms (using NOT terms)	April 26, 2023, returned 466 records	August 23, 2024, returned 239 records	“Testosterone” AND “female” AND “athlete”	*P*-values, control groups, measures of serum and/or salivary T levels	Review articles, hormone therapy articles, combined sex analyses only, studies of males only, and transexual men or women only
Table 5	“PCOS” and the results of “strength” “muscularity” and “athleticism” (using OR terms)	September 19, 2023, 165 records	August 26, 2024, returned 51 records	“PCOS” AND “strength” OR “muscularity” OR “athleticism”	*P*-values, control groups, correlations between PCOS and measures of either strength, muscularity, or athleticism	Review articles and studies of transexual females
Table 6	“Behavioral dominance” (using the AND term) with the results of “testosterone” and “digit ratio” (using OR terms), while excluding the results of “male” “men” “man” “boy” terms (using NOT terms)	October 5, 2023, 361 records	August 26, 2024, 30 records	“Testosterone” AND “dominance” AND “female”	*P*-values and correlations between serum T or digit ratio and behavioural dominance	Review articles, combined sex analyses only, studies of only men, and transexual men or women only
Supplementary Table S1	“Neolithic” or “ancestral” (using the OR term) with the results of “female*” or “women” (using the AND term). Then, the previous terms were combined (using the AND term) with the term's “strength” “muscularity” “bone mineral density” “heavy labor” (using OR terms)	October 12, 2023, 84 records	August 28, 2024, 92 records	“Neolithic” OR “ancestral” AND “female” OR “women” AND “strength” OR “muscularity” OR “heavy labor” OR “bone mineral density”	*P*-values or correlations between groups of ancestral, neolithic, or modern females and measures of either strength, muscularity, BMD, or heavy labor	Review articles, combined sex analyses only, and studies of only men
Supplementary Table S3	“Athletic performance” and “athlete*” (using the OR term) with the results of “digit ratio” (using the AND term)	October 16, 2023, 55 records	August 28, 2024, 60 records	“Athletic performance” OR “athlete*” AND “digit ratio”	*P*-values and correlations between athletic performance and digit ratio	Review articles, combined sex analyses only, studies of men only, and transexual men or women only
Supplementary Table S4	“Testosterone” and “PCOS” (combined using the AND term) with “overweight” and “obese” (using the OR term)	October 25, 2023, 919 records	N/A	N/A	Mean serum T levels for both obese or overweight females with PCOS and controls	Review articles and articles of transexual women only
Supplementary Table S5	“Testosterone”, “PCOS” and “lean” (combined using the AND term)	October 25, 2023, 202 records	N/A	N/A	Mean serum T levels for both lean females with PCOS and controls	Review articles and articles of transexual women only
Supplementary Table S7	“PCOS” and the results of “bone mineral density”	September 25, 2023, 100 records	August 29, 2024, 86 records	“PCOS” AND “bone mineral density”	*P*-values, control groups, correlations between PCOS and measures of BMD	Review articles and studies of transexual women only
Supplementary Table S8	“Testosterone” and “bone mineral density*” (using the AND term), with the results of “female”, “woman”, “women” and “girl” (using the OR term), while excluding the results of “male” “men” “man” “boy” terms (using NOT terms)	November 29, 2023, 332 records	August 31, 2024, 40 records	“Testosterone” AND “bone mineral density*” AND “female”	*P*-values, correlations between serum or salivary T and BMD in healthy females	Review articles, hormone therapy articles, combined sex analyses only, studies of only males, transexual men only, and transexual women only
Supplementary Table S9	“Digit ratio*” and “anogenital distance*” (using the OR term) with the results of “bone mineral density” (using the AND term)	December 6, 2023, 19 records	August 31, 2024, 15 records	“Digit ratio*” OR “anogenital distance*” AND “bone mineral density”	*P*-values, females only, correlations between 2D:4D or AGD with BMD	Review articles, combined sex analyses only, studies of males only, transexual men only, and transexual women only

Web of Science (WOS), PubMed, and Google Scholar databases were searched for each relevant table. Google Scholar, a more broadly inclusive search engine, was searched using the specific terms listed in Table 1, for any date, and with sorting by relevance. For this database, the first 200 citations were included in the initial screening for each relevant table. The reference lists of relevant articles from each search were also searched for additional relevant citations. In each table that analysed serum or salivary testosterone concentrations, these concentrations were converted to nmol/litre (nmol/L) to standardize across studies.

## Results

### Markers of prenatal testosterone and athleticism, strength, muscularity, dominance, and bone mineral density in healthy females

High prenatal testosterone has been strongly associated with PCOS in animal models (39, 40), and prenatal testosterone levels can be indexed by measuring AGD or, with considerably less accuracy and repeatability, 2D:4D (41–43). By the hypothesis addressed here, lower 2D:4D and higher AGD (and thus higher inferred prenatal testosterone) should be associated with higher strength, athleticism, muscularity, bone mineral density, and dominance in females.

In the data reviewed here, lower 2D:4D ratios were significantly associated with measures of higher athleticism, speed, strength, or endurance across 24 of 36 studies overall (Supplementary Table S3). However, studies regarding correlations between measures of athleticism, strength, muscularity, dominance, or BMD and AGD were not found in the literature search. There was limited data regarding associations between markers of prenatal testosterone and dominance. However, Manning and Fink (44) reported a significant negative association between right 2D:4D and dominance scores in females, where dominance scores were measured via questions from the International Personality Pool (IPIP). Digit ratios were significantly and positively associated with measures of BMD (indicating an association of BMD with lower prenatal testosterone) in two of three studies (Supplementary Table S9).

### Serum or salivary testosterone and athleticism and its correlates in healthy females

By the main hypothesis addressed here, athleticism and its correlates should be associated with higher adult testosterone, among healthy females without diagnoses of PCOS. Higher testosterone levels have been significantly and positively associated with measures of athletic performance across 20 of the 34 studies in Table 2. The various measures of athletic performance in Table 2 included strength, sprint speed, stamina, lean muscle mass, training intensity and motivation, muscle fatigue, and competitiveness, in the contexts of a wide variety of sports and athletic training.

**Table 2:** Serum and salivary testosterone levels in relation to female athletic performance.

Participants	Findings (significant refers to *p* < 0.05)	Reference
18 amateur female runners	Serum T concentrations were significantly negatively correlated with muscle fatigue after ultra-endurance events	(45)
12 elite female adolescent volleyball players	Serum T levels decreased significantly after exercise resistance training compared to pre-training levels	(46)
599 Russian international-level female athletes and 298 controls	Serum T levels were significantly positively associated with athletic success in sprinters, but not in endurance or mixed-type athletes	(47)
24 young physically active females and 24 matched controls	Females given 10-mg of T cream daily for 10 weeks significantly improved their aerobic performance, but their anaerobic performance did not differ significantly from the control group	(48)
9 elite and 21 non-elite female athletes	Salivary T concentrations were significantly positively correlated with competitive desire and training motivation in both groups	(49)
30 female volleyball players	Both cluster set and traditional set resistance training groups had significantly higher serum T levels than the control group following an 8-week training program	(50)
26 females and 26 boys competing in an Olympic weightlifting competition	Pre-competition serum T was significantly negatively correlated with weightlifting performance after body mass was controlled	(51)
31 female athletes from various sports clubs and 21 female ballet dancers	Females with higher levels of T (T > 50 ng/dl) began training at a significantly earlier age and their training period was significantly longer compared to females with lower levels of T (T < 10 ng/dl)	(52)
19 elite female basketball players	No significant changes in salivary T concentrations were observed from pre-training to post-training across the 4 sampling points	(53)
71 post-menopausal females	Females who received weekly injections of 25-mg T enanthate for 24 weeks showed a significant increase in chest-press power, loaded stair power and lean body mass compared to the placebo group	(54)
12 elite female netball players	Salivary T levels were significantly positively correlated with bench press and squat, as well as maximal medicine ball throw	(55)
31 healthy females and 14 healthy males	There were no significant changes between pre-exercise and post-exercise salivary T concentrations in females performing elbow flexor resistance exercise workouts	(56)
19 adult elite powerlifters (8 males and 11 females)	No significant changes were found in T concentrations between all competition phases for females	(57)
18 competitive female swimmers and 18 controls	Serum T levels in female swimmers were significantly higher in the swimmer group compared to the control group	(58)
14 elite female basketball players	There were no significant changes in salivary T concentrations across endurance, strength and power resistance training schemes when compared to pre-exercise values	(59)
12 elite female basketball players	The changes (*Δ* Pre-Post training) in strength and salivary T concentrations were significantly positively correlated at 0,730 h, although training itself had no significant effect on T concentrations	(60)
20 Taekwondo athletes (10 females and 10 males)	Changes in serum T levels were not significant between pre and post Taekwondo fighting simulation in female participants	(61)
27 (13 females and 14 male) elite national level volleyball players	A one-hour volleyball practice led to significant increases in serum T compared to pre-practice levels in females	(62)
22 female and 48 male elite athletes	Countermovement jump height was significantly positively correlated with T levels in female athletes. T levels and vertical jumping ability in female sprinters were significantly higher than those of female volleyball players	(63)
30 female collegiate tennis athletes	Resting serum concentrations of T increased significantly during both periodized and non-periodized resistance training over the 9-month period	(64)
Track and field sprinters (*N* = 6 male and *N* = 6 female)	After the training session, there were no significant changes in the levels of serum T in female sprinters	(65)
11 pre-menopausal women with fibromyalgia and 12 sedentary healthy control women	No significant changes were observed in serum T and free T during the heavy resistance fatiguing loading either during the pre-training or post-training conditions	(66)
14 female collegiate distance runners and 14 controls	No significant changes were observed in resting serum T levels after resistance training interventions	(67)
10 sedentary females in the training group and 10 in the control	After 12 weeks of resistance training program, the training group showed a significant increase in serum T compared to the control group	(68)
9 healthy females	Heavy-resistance exercise protocols did not significantly alter serum concentrations of T	(69)
12 females in single-set circuit group (SSC), 12 in multiple-set (MS), and 10 in control	Increases in serum T were observed for both SSC and MS after 12 weeks, but only MS showed a significant increase at 24 weeks of training	(70)
51 females aged 49 to 74 years	Strength training increased serum T after the first half of training, but these returned to baseline values at the end of the entire training period (21 weeks)	(71)
28 females over 65 years of age	No significant changes were observed in total serum T levels after the 3-month walking exercise program	(72)
45 female and 23 male young professional athletes	No significant correlations were found between serum T levels and measures of power, speed, body composition in young female athletes	(73)
106 female Olympic athletes and 117 sedentary controls	There were no differences in serum T levels between female athletes and controls	(74)
335 female sprinters, 106 throwers, and 141 long distance runners	Female athletes involved in sprinting and throwing activities showed significantly higher serum T levels than females involved in long-distance running	(75)
26 female and 45 male junior athletes	Serum T significantly increased across the simulated weightlifting competition in females, but not salivary T	(76)
16 cross-trained healthy pre-menopausal females	Serum T levels after an endurance exercise session were significantly higher than the resting control session, and serum T levels after resistance exercises were higher but not significant	(77)
1,332 elite female athletes and 795 elite male athletes	Females with higher serum free T levels performed significantly better in 400 m, 400 m hurdles, 800 m, hammer throw, and pole vault compared to females with lower serum free T levels	(78)

T, testosterone; *N*, sample size.

For studies that quantified serum testosterone levels, four of nine showed significantly higher testosterone levels in athletes compared to controls (Table 3). Across the studies that quantified salivary testosterone levels, all four showed significantly higher mean testosterone levels in athletes than in controls (Table 3).

**Table 3:** Comparison of serum and salivary testosterone levels between healthy female athletes and female controls/non-athletes.

Population	Method of T quantification	Athlete T (nmol/L) and *P* values	Control T (nmol/L)	Reference
22 Olympic-level field hockey athletes and 87 university participant pool non-athletes of the age range 18–25 years	EIA (saliva)	0.145 ± 0.054 (*P* = 0.006)	0.103 ± 0.054	(79)
24 soccer referees and a control group of 24 non-athletic females of the age range 18–23 years	IA	T0 (resting levels) = 0.49 ± 0.01, T1 = 1.98 ± 0.13, T2 = 1.63 ± 0.13, T3 = 1.16 ± 0.09 (*P* < 0.0001 T0-T1 and T1-T2, *P* < 0.001 for T2-T3 and T3-T0 compared to control)	Differences between T0 and all other time points were N.S. within controls. No T values were given	(80)
94 elite athletes (mean age 25 years) and 86 untrained controls (mean age 26 years)	LC-MS-MS	1.0 ± 0.37 (*P* > 0.05)	0.99 ± 0.40	(81)
599 Russian international-level athletes (aged 16–35 years) and 298 age-matched controls	EIA	1.65 ± 0.87 (0.08–5.82) (*P* = 0.057) *No SD reported	1.76 ± 0.06 (0.38–2.83) *No SD reported	(47)
6 elite and 16 non-elite athletes with a mean age of 21 years	RIA (saliva)	0.13 ± 0.08 (*P* < 0.05)	0.06 ± 0.03	(82)
9 elite athletes and 21 non-elite. The mean age was 20 years between both groups	EIA (saliva)	0.1, 0.2, and 0.1 for elite athletes in the Follicular, Ovulatory, and Luteal phases, respectively (*p* < 0.05)	0.075 for non-elites across all phases	(49)
106 Swedish Olympic athletes and 117 age and BMI-matched sedentary controls. The mean ages of athletes and controls were 26 years	LC-MS-MS	0.99 ± 0.35 (*P* > 0.05)	0.99 ± 0.39	(74)
25 young elite swimmers and 21 control subjects with a mean age of 15 years	RIA	High T swimmers’ group = 2.20 ± 0.59, low T swimmers’ group = 1.25 ± 0.24 (*P* < 0.001 for high T vs. control group, *P* = N.S. for low T vs. control group)	1.32 ± 0.49	(83)
9 elite international competitor athletes and 9 non elite athletes, the average ages were 25 years for elites and 23 years for non-elites	EIA (saliva)	0.302 (*P* < 0.001) *No SD reported	0.142 *No SD reported	(84)
18 adolescent swimmers and 18 control subjects. The mean age was 15 years	RIA	1.9 ± 0.7 (*P* < 0.005)	1.4 ± 0.4	(58)
15 eumenorrheic adolescent endurance athletes and 16 non-athletic controls, the mean age was 15 years	RIA	1.01 ± 0.30 (*P* > 0.05)	0.87 ± 0.29	(85)
8 athletes in endurance sports aged 16–35 years and 8 females in the control group	RIA	0.60 ± 0.20 (*P* > 0.05)	0.70 ± 0.20	(86)
15 endurance athletes, where 9 were in the experimental training group and 6 were in the control group	FI	3.5 ± 0.5 (*P* < 0.05)	2.4 ± 0.6	(87)

T, testosterone; BMI, body mass index; SD, standard deviation; EIA, enzyme-linked immunoassay; RIA, radioimmunoassay; FI, fluoroimmunoassay; LC-MS-MS, liquid chromatography tandem mass spectrometry; IA, immunoassay.

Across the eight studies that quantified serum testosterone levels and included control groups, mean serum testosterone levels were approximately 18% higher in the athlete group compared to controls. Across the four studies that analyzed salivary testosterone levels and included control groups, mean salivary testosterone levels were about 90% higher in the athlete group compared to healthy controls (Table 4 and Supplementary Table S2). The studies reviewed here did not compare levels of testosterone from different sources (mainly serum vs. saliva). High salivary testosterone levels compared to serum can be influenced by several factors, including binding proteins in serum (e.g., SHBG and albumin), salivary gland function, measurement differences in sensitivity or specificity, and physiological variations such as stress, circadian rhythms, or individual metabolic differences (88).

**Table 4:** Comparisons of serum and salivary testosterone percent difference between controls and women athletes, women with overweight PCOS, and women with lean PCOS.

Group	*N* (number of studies)	Mean percent difference (SD)
Athlete vs. control (serum T)	8	18.1 (26.7)
Athlete vs. control (salivary T)	4	90.1 (30.2)
Overweight PCOS vs. control (serum T)	17	83.8 (50.2)
Lean PCOS vs. control (serum T)	12	71.3 (44.3)
All BMI PCOS vs. control (serum T)	29	78.6 (48.2)

PCOS, polycystic ovary syndrome; T, testosterone; BMI, body mass index; SD, standard deviation.

The mean percent difference and the standard deviation (SD) were calculated based on the average percent difference value and SD across each respective group (for female athletes see Supplementary Table S2, for overweight/obese females with PCOS see Supplementary Table S4, for lean females with PCOS see Supplementary Table S5). The form of testosterone measured in each group is shown in brackets (e.g., serum T or salivary T).

### Serum and salivary testosterone levels in relation to measures of BMD in pre-menopausal and post-menopausal healthy females

By the hypothesis addressed here, higher serum and salivary testosterone levels should be associated with increased BMD in healthy females. Serum testosterone was significantly positively associated with BMD in 12 of 30 studies (Supplementary Table S8). There were no significant associations between testosterone and BMD in 14 studies, and in 2 studies there were negative correlations. There was also a mix of positive and null results in 2 of 25 studies: for example, Nunes et al. (89) found that serum testosterone levels were significantly positively associated with BMD scores for the hip, but not for the lumbar spine, in postmenopausal females. This diversity of results was apparently not related to pre- vs. post-menopausal status, as similar patterns of positive and null results were found in each group (Supplementary Table S8). Salivary testosterone was significantly positively associated with BMD in the single study that analyzed this metric (90). These findings suggest that testosterone and BMD are positively correlated, but with notable heterogeneity across studies, perhaps due in part to the high diversity of the populations and skeletal regions subject to analysis.

### Testosterone levels, muscle mass, physical strength, endurance, and BMD in females with PCOS

The results presented above that modest (15%–20%) increases in serum testosterone are linked with enhanced athleticism in healthy females raises the question of whether females with PCOS, who show notably larger increases in testosterone (91–93), benefit in terms of strength, muscularity, and athleticism.

By the hypothesis addressed here, females with PCOS are predicted to exhibit increased muscle mass, physical strength, and endurance compared to healthy matched controls, predominantly as a function of their increased levels of testosterone. This hypothesis was largely supported by available data from the literature (Table 5). Overall, females with PCOS exhibited increased muscle mass (seven of eleven studies), physical strength (six of eight studies), sporting performance (one of one), and endurance (one of three studies) when compared to healthy controls. All the other studies showed no significant differences between groups.

**Table 5:** Athletic performance, strength, and muscularity in females with PCOS.

Participants	Findings (significant refers to *p* < 0.05)	Reference
31 females with PCOS (mean age 27) and 13 controls (mean age 30)	The PCOS group demonstrated significantly higher cardiorespiratory fitness (VO_2_ max), handgrip strength, and exercise capacity compared to controls	(94)
70 females with PCOS (mean age 28) and 93 controls (mean age 29)	The PCOS group had significantly greater total lean mass in the dominant hand (but a non-significant difference in the non-dominant) and significantly greater hand grip strength in both the dominant and non-dominant hands compared to controls	(95)
44 females with PCOS (mean age 21) and 32 BMI-matched controls (mean age 22)	Females with PCOS showed significantly increased average lower limb power (measured by knee extension and flexion) compared to controls	(96)
73 females with PCOS (mean age 28) and 97 controls (mean age 29)	Females with PCOS in a progressive resistance training intervention study showed a significant increase in maximum strength on the bench press, leg extensions, and arm curl compared to non-PCOS females for all exercises	(91)
10 females with PCOS (mean age 25) and 10 controls (mean age 24)	There was no significant difference in pelvic floor muscle thickness between females with PCOS and healthy females	(97)
42 females with PCOS (mean age 25) and 13 premenopausal controls (mean age 26)	Females with PCOS showed significantly higher measures of muscle tone and maximum voluntary contraction of pelvic floor muscles compared to controls	(98)
33 females with PCOS (mean age 27) and 39 controls (mean age 30)	There were no significant differences in pelvic floor muscle thickness or muscle activity between PCOS females and controls	(99)
45 sedentary females with PCOS (mean age 28) and 52 controls (mean age 29)	Females with PCOS showed significantly increased muscle mass and total lean mass compared to controls after progressive resistance training for 4 months	(100)
40 females with PCOS (mean age 26) and 40 controls (mean age 28)	Females with PCOS showed significantly higher bench press strength, muscle strength relative to lean muscle mass in the dominant lower limb and isometric handgrip strength compared to the control group	(101)
36 females with PCOS (mean age 27) and 43 controls (mean age 30)	There was no significant difference in mean pelvic floor muscle strength between the PCOS group and controls	(102)
30 females with classic PCOS (mean age 23), 13 with ovulatory PCOS (mean age 23), and 22 controls (mean age 27)	Total and trunk lean mass were significantly higher in the PCOS group in comparison to the ovulatory PCOS group and the control group. Arm and leg lean mass were not significantly different between the groups	(103)
25 postmenopausal females with PCOS aged 61–78 and 68 randomly allocated age-matched controls (no mean ages reported)	There was no significant difference in lean mass between PCOS females and controls	(104)
95 females with PCOS (mean age 24) and 90 controls (mean age 24)	Lean muscle mass was significantly higher in females with PCOS compared to weight-matched controls	(105)
10 overweight and obese females with PCOS (mean age 33) and 16 age-and weight matched controls (mean age 36)	There was no significant difference in maximal aerobic capacity (VO_2_ max) in overweight females with PCOS compared to overweight non-PCOS controls	(106)
37 females with PCOS and 35 controls (no mean ages reported)	There were no differences in hand strengths in dominant and non-dominant hands between the two groups	(107)
10 lean females with PCOS (mean age 24) and 10 controls (mean age 23)	Lean females with PCOS showed significantly lower total lean mass values compared to controls	(108)
59 females with classic PCOS, 23 females with ovulatory PCOS, and 51 controls (median age 28)	Mean appendicular lean mass index, lumbar spine, and total femur BMD were significantly higher in females with classic PCOS than control	(109)
34 females with PCOS (mean age 43) and 32 female controls (mean age 42)	Non-obese PCOS females had higher lower extensor muscle strength than controls and obese PCOS females had higher total abdominal muscle area than controls. All other measures of muscularity did not differ between females with PCOS and controls	(110)
40 female athletes with PCOS and 40 healthy female athletes (mean age 24)	Sporting performance was significantly higher in females with PCOS compared to controls	(111)
9 females with PCOS (mean age 33) and 9 controls (mean age 29)	There were no significant differences in training intensity and VO_2_ max between females with PCOS and controls	(112)

PCOS, polycystic ovary syndrome; BMI, body mass index.

In contrast to these results for various measures of strength and muscularity, the differences in BMD between females with and without PCOS varied considerably. Across 21 studies, six showed significantly higher BMD in females with PCOS compared to controls, six showed significantly lower measures of BMD in females with PCOS, and 10 showed no significant differences between the two groups (Supplementary Table S7). These findings indicate that PCOS cannot be considered to involve higher BMD, for reasons that remain unclear but may involve insulin resistance (113, 114) or other factors.

### Comparison of mean total serum testosterone levels across female athletes, females with PCOS, and controls

Are the high testosterone levels in females with PCOS comparable to the levels found in female athletes? Although direct comparison of testosterone levels between female athletes and females with PCOS cannot be conducted using data from the literature (due to methodological differences in testosterone quantification methods across studies), the percent differences in serum testosterone levels between females with PCOS or athletes, and each respective control group can usefully be compared. Using this approach, females with PCOS had an average of about 79% higher serum testosterone levels compared to controls across a sample of studies (mean = 78.6, SD = 48.2, *N* = 29), whereas female athletes showed a smaller difference (about 18%) in serum testosterone levels compared to their respective controls (mean = 18.1, SD = 26.7, *N* = 8) (Table 4).

**Figure 1:**
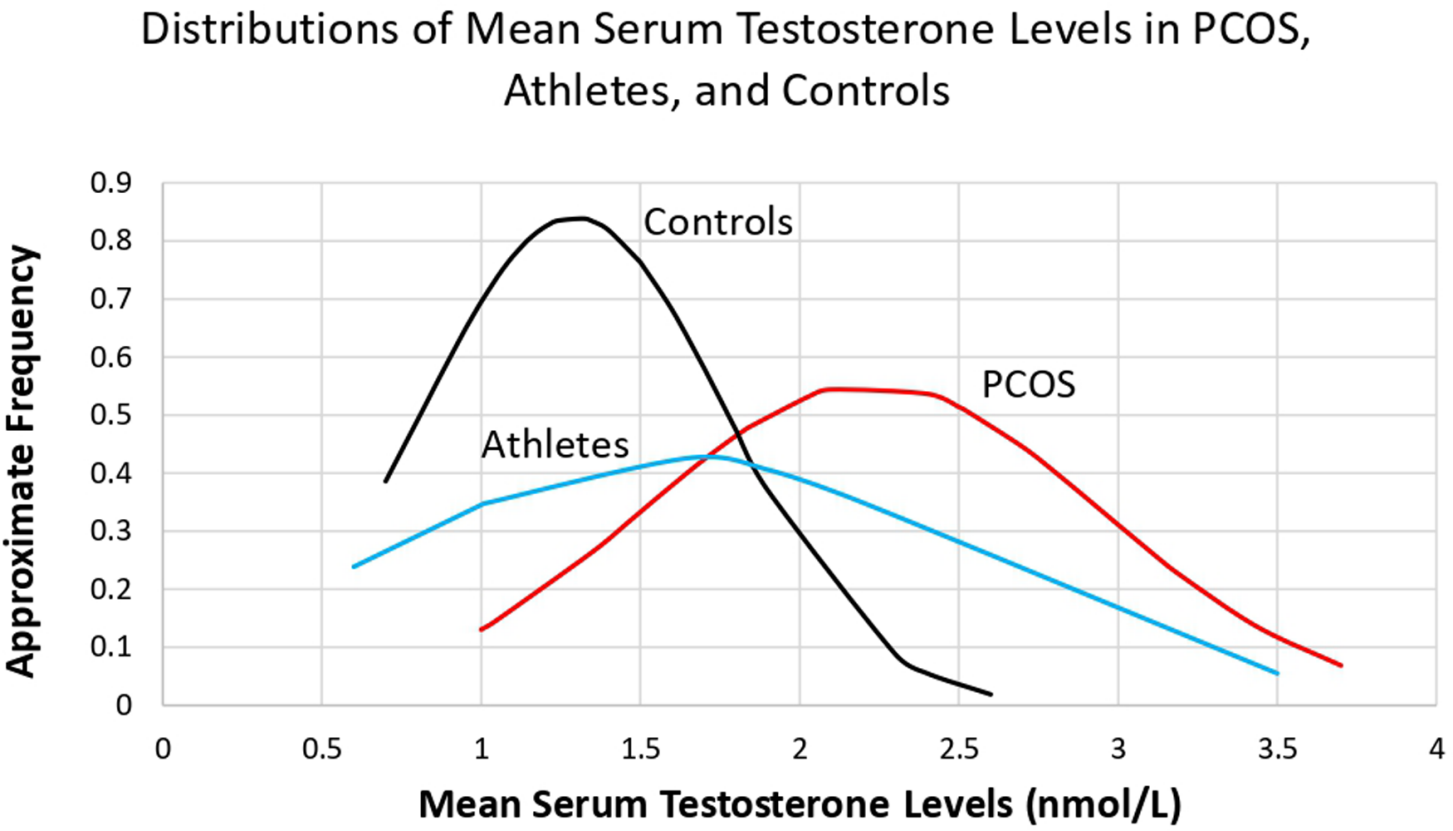
Comparison of the distributions of mean serum testosterone levels in women with PCOS, athletes, and controls, calculated from the data in Supplementary Table S2, Supplementary Table S4, and Supplementary Table S5.

Given these simple mean differences, are the distributions of mean total serum testosterone levels comparable, and overlapping, between female athletes, controls, and females with PCOS? Mean serum testosterone levels, calculated from the data in Supplementary Table S2, Supplementary Table S4, and Supplementary Table S5 were 1.61 nmol/L (*N* = 8, SD = 0.94, range = 0.6–3.5) in the athletes, 1.29 nmol/L (*N* = 37, SD = 0.48, range = 0.5–2.6) in the controls, and 2.23 nmol/L (*N* = 29, SD = 0.72, range = 1–3.7) in females with PCOS. The data showed an approximately normal distribution in the PCOS group, as well as considerable overlap between all three groups (Figure 1). Athletes and controls did not show normal distributions, likely due to the lower end of the distribution not falling below the normal range of testosterone in healthy females (range = 0.52–1.7 nmol/L (115). The athlete group exhibited an especially broad overlap with the PCOS group. This overlap was notably different from that of the overlap with the control group, especially in the region where mean serum testosterone levels exceeded 2.5 nmol/L. It is important to note that the data presented here is from Western and modern populations, and that testosterone levels are probably lower in females from small-scale societies (116).

### Strength, muscularity, and athleticism in lean females with PCOS

PCOS is commonly associated with high BMI and insulin resistance (117–119), which appears to be due, in part, to the modern obesogenic environment characterized by low physical activity and high-energy food intake (9). So-called “lean” females with PCOS exhibit BMIs near or below 25 kg/m^2^, and as such, these females may provide a relatively more-suitable model (compared to high-BMI females) of PCOS-related traits as found in traditional or ancestral populations.

By the hypothesis addressed here, lean females with PCOS are predicted to exhibit increased strength, muscularity, and athleticism compared to healthy matched controls, due at least in part to higher testosterone. The elevations of testosterone levels in lean females with PCOS (mean = 71.3, SD = 44.3, *N* = 12) are similar to those of overweight females with PCOS (mean = 83.8, SD = 50.2, *N* = 17; Table 4). Only one study (not included in the previous section or in Table 5) has reported muscular strength in lean females with PCOS compared to controls (96); these authors found that females with PCOS exhibited increased average power in the lower limbs, and that this measure of performance was positively correlated with levels of bioavailable testosterone. Peak muscle force output was also higher in females with elevated BMI, in both controls and females with PCOS.

Despite the paucity of data on lean females with PCOS, there is considerable evidence showing a high prevalence of PCOS-related traits in female athletes, who are typically lean. Thus, PCOS was found to be the main cause of menstrual disorders among Olympic female athletes, where the incidence of polycystic ovaries in athletes who were not using hormonal contraceptives was high (37%) (120) compared with the estimated incidence of approximately 20% in the general population (121). Dadgostar et al. (122) reported that PCOS was the most common cause of menstrual irregularities among 788 Iranian female athletes, where 9% of individuals had amenorrhea or oligomenorrhea. Coste et al. (58) found that 11 out of 18 female swimmers had serum testosterone levels >0.5 ng/ml and were thus classified as hyperandrogenic, and 45% of these hyperandrogenic swimmers met the Rotterdam criteria for PCOS (two or three of the three symptoms hyperandrogenism, oligo- or anovulation, and polycystic ovaries). Moreover, Rickenlund et al. (123) found that one third of female endurance athletes with menstrual irregularities showed hyperandrogenism. These findings indicate that there is a high prevalence of PCOS, and PCOS-related traits, in female athletes.

Interpretation of these data is predicated on distinguishing between two distinct causes of reduced ovulation rates in lean female athletes (124–126). First, amenorrhea in athletes with low BMI may be caused by energy deficiency, specifically low-fat reserves, which cause hypothalamic inhibition through reduction of GnRH secretion (127, 128). Second, oligomenorrhea in female athletes has been found to be associated with hyperandrogenism, as in females with PCOS (86, 123, 129). These two groups exhibit distinct hormonal profiles, with the main distinguishing trait being 24-hr secretion of testosterone (86). The generally elevated testosterone levels of female athletes, documented above, suggests that they fall predominantly into the latter group.

### Social and physical dominance among females with higher testosterone

In nonhuman female primates, social and physical dominance are consistently associated with higher testosterone levels (130–135). By the hypothesis addressed here, both females with higher testosterone levels (without PCOS), and females with PCOS, are predicted to show high social and physical dominance compared to healthy matched controls.

Across 15 studies measuring testosterone levels in relation to social and physical dominance in females, measures of dominance were significantly and positively correlated with testosterone levels in 10 studies (Table 6). Across these studies, dominance was typically measured by acts of aggression, intimidation, forcefulness, frequency of smiling, or gaze duration. Human gaze is an important indicator of dominant and submissive behaviors, where longer durations of face-gazing is indicative of higher dominance (49, 138, 143). Significant negative relationships have also been reported between right 2D:4D digit ratio and dominance scores (44), as well as between 2D:4D and dominance ratings (150), which suggests that higher prenatal testosterone is associated with dominance. There are apparently no studies in which measures of social or physical dominance in females with PCOS are compared to controls.

**Table 6:** Serum or salivary testosterone levels in relation to measures of behavioral dominance in females.

Participants	Findings (significant refers to *p* < 0.05)	Reference
19 females, aged 18–25 years	Participants administered 0.5 mg T showed a significantly larger peri-personal space preference around the self than placebo	(136)
337 adolescents, 192 females, mean age: 15	There were no significant correlations between salivary T and measures of dominance in females	(137)
26 female athletes from different sports, mean age: 22	A positive relationship between salivary T reactivity and gaze duration was observed when losing the coin toss game	(49)
18 females with social anxiety disorder and 19 controls, with mean ages of 23 and 25 respectively	Participants with social anxiety disorder who received 0.5 mg T showed significantly alleviated gaze avoidance when compared to placebo	(138)
82 females, mean age: 21	Females showed a significant positive correlation between T levels and self-reported dominance. Dominance scores were measured by scores to a questionnaire containing basic demographic items and the IPIP scale for social dominance	(139)
92 females, mean age: 26	Trait dominance was not significantly associated with serum T levels	(140)
54 females, mean age: 21.6	There were no significant differences in trait dominance and baseline T between the T and placebo groups	(141)
24 females, mean age: 29 years	Participants showed significantly diminished avoidance of angry faces when administered 0.5 mg T compared to the placebo group	(142)
20 females aged 20–25 years	After sublingual administration of 0.5 mg T, gaze aversion from angry faces was significantly slower than gaze aversion from happy faces compared to the placebo group	(143)
53 female graduate and undergraduate students, mean age: 20	There was no significant relationship between basal salivary T and “n Power” (implicit power motivation/dominance over others) in single females, but there was a trend negative correlation between basal T and “n Power” for females in close relationships	(144)
16 females aged 19–25 years	Participants who were sublingually administered 0.5 mg T showed significantly accelerated cardiac responses to angry faces compared to participants who received the placebo. This effect was considered a likely indicator of aggressiveness and dominant behavior	(145)
52 s year female medical students, mean age: 20	Dominance scores on the Simple Adjective Test (a questionnaire designed to measure dominance) were significantly positively correlated with serum T levels	(146)
87 female inmates, mean age: 33	Aggressive dominance was significantly positively correlated with T levels. Dominance was measured by institutional behavior prison records and interviews with staff members	(147)
34 female university students, aged 19–26 years	The number of scans in which subjects were smiling was negatively correlated with T levels. Infrequent smiling was considered as a behavior that was indicative of dominance	(148)
10 dominant and 10 submissive female undergraduate students, mean age: 18.5	Serum T levels were not significantly different between the dominant and submissive groups. These two groups represented the top and bottom quintiles from scores on the Dominance subscale of the Adjective Check List	(149)

T, testosterone; IPIP, International Personality Item Pool.

### Females in ancestral environments showed evidence of substantial strength and muscularity

A systematic review of archaeological evidence related to muscularity in females demonstrated five main lines of evidence suggesting that females in some ancestral populations exhibited high strength and muscularity, in the contexts of their local ecologies (Supplementary Table S1).

First, cross sectional geometry (CSG) analysis of humerus bones showed that Muisca females from the Tibanica archaeological site (1,000–1,400 AD) in Colombia had highly robust and strong upper arms, and for six of eight CSG measures they exhibited significantly larger CSG values than their male peers (151). Macintosh et al. (152) also reported that Neolithic, Bronze Age, and Iron Age females had notably strong upper limbs with CSG properties closely matching the values observed in modern semi-elite female rowers. Robust upper bodies and strengthened humeri were also documented in Pottery Mound (700–500 YBP) females from New Mexico, USA (153). Kralick and Zemel (154) reported that young female skeletons from the agricultural period (1,150–1,550 AD) from the Georgia coast had significantly lower CSG values for nearly every measurement when compared to pre-agricultural (2,200 BC–AD 1,150) young adults from the same site. By contrast, Marchi et al. (155) reported that the CSG properties of Later Upper Paleolithic and Neolithic (6,000–5,500 BP) indicated patterns of decreased robusticity in females.

Second, BMD analyses conducted in Norway showed that young medieval females (1,050–1,536 CE) had the highest mean BMD of all time periods that were studied (750 CE–present), including modern females (156). Similarly, Holck (157) reported that females from the medieval period had significantly higher BMDs when compared to modern females, but there were no significant differences in BMD when comparing Prehistoric (5,000 BC–800 AD) or Viking Age (800–1,050 AD) females to modern females. Moreover, Spinek et al. (158) found that Neolithic females showed significantly higher BMD values compared to early medieval, medieval, and modern females.

Third, most (about 60%–70%) of studies that quantified musculoskeletal stress marker (MSM) scores (that indicate muscularity) have reported that males exhibited higher MSM scores than females, on average. However, several studies showed opposite sex-related patterns, where females exhibited higher MSM scores than males. For example, MSM scores from skeletons from both Natufian and Neolithic sites in the Levant (8,000–6,000 BC) were higher in females than in males, particularly in muscles attached to the ulna or radius (159). Similarly, using a sample of 185 skeletons from Pecos Pueblo (1,200–1,838 AD) in New Mexico, Chapman (160) reported that females had higher MSM scores in the pectoralis minor, a muscle likely utilized in grinding maize. Within a sample of 136 Early Thule Eskimo skeletons from northwest Hudson Bay (radiocarbon dated to 1,205 AD), Hawkey and Merbs (161) found that females had higher MSM scores for the trapezius and pectoralis minor, muscles likely utilized in rowing umiaks. Moreover, Hershkovitz and Gopher (162) reported that MSM scores in Neolithic females were significantly higher than in the preceding Natufian populations, which suggests that Neolithic females took over a greater proportion of physical activities compared to Natufian females.

Fourth, anthropological evidence suggests that females in some ancestral environments engaged in strenuous physical activities and heavy agricultural labor. For example, Miller et al. (151) proposed that stronger humeri in skeletal samples of Muisca females from the Tibanica site in Colombia (1,000–1,400 AD) was due to regular involvement in vigorous physical activities, such as grinding maize. Similarly, Cassidy (163) reported that in archaic-period (3,000–1,000 BC) Indian Knoll populations, females were exposed to an increased workload with the adoption of agriculture. Moreover, Bridges (164) reported that maize agriculture was more physically demanding than hunting and gathering and affected females more than males. Finally, Weber and Bettinger (165) reported that Late Neolithic to Early Bronze Age (5,800–4,000 BP) males and females showed non-significant differences in femur robusticity and a relatively equitable distribution of labor. It is important to note that the anthropological studies selected in this review describing physical activities and labor do not necessarily discuss “strength”, but more so describe “use”. As such, studies that measure BMD, CSG, or MSM provide clearer evidence regarding strength and muscularity in females from ancestral environments.

Fifth, a recent study provided evidence that the majority (about 80%) of females, from 63 different foraging societies across the globe, have been documented to participate in hunting over the last 100 years (166). The 63 societies described included 19 from North America, six from South America, 12 from Africa, 15 from Australia, five from Asia and six from the Oceanic region. Anderson et al. (166) also reported that over 70% of hunting done by females was described as intentional, indicating that females are skilled in the practice and exhibited distinct hunting strategies and training regimes compared to their male counterparts [e.g., (167–170)]. This study provides evidence that females in foraging societies across the world participate in hunting during recent time periods, thus providing another line of evidence for the proposed benefits of increased testosterone within these societies, at least to the degree that increased strength and athleticism enhance hunting abilities.

Taken together, this evidence suggests that high levels of strength and muscularity were not uncommon among females in some ancestral environments, thus representing socioecological contexts for benefits to relatively high testosterone.

## Discussion

**Figure 2:**
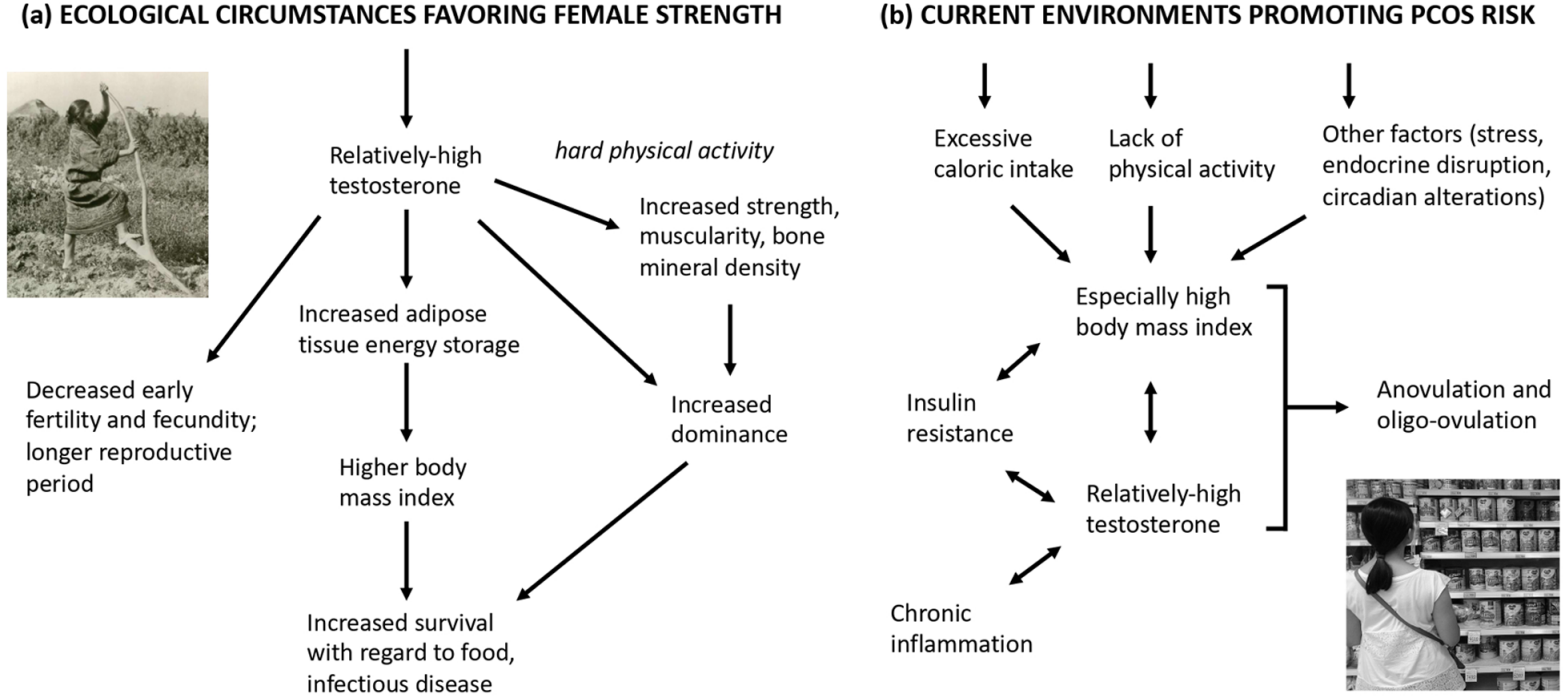
Hypothesized ecological circumstances favoring **(a)** high testosterone in females, compared to **(b)** current environments promoting PCOS risk. The single-headed arrow represents hypothesized effects, while the double-headed arrow represents positive associations between traits.

In this study we have reviewed diverse lines of evidence to evaluate the hypothesis that the risks, symptoms, and correlates of PCOS in current populations represent, in part, extreme and maladaptive manifestations of adaptive traits, especially phenotypes associated with relatively high testosterone levels, that appear to confer benefits to females in terms of muscularity, athleticism, dominance, and robustness. Overall, this hypothesis, as set forth in various ways by Azziz et al. (16), Casarini et al. (171), Ünlütürk et al. (18), Fessler et al. (17), Charifson and Trumble (9), Dumesic et al. (36), and Parker et al. (11), was supported by multiple convergent lines of evidence. This evidence includes: (1) the links of markers of prenatal testosterone with both PCOS and athleticism, (2) the positive associations of serum testosterone with strength in healthy females, (3) the higher strength and muscularity found in females with PCOS, (4) the links of testosterone with higher dominance in females of humans and other mammals, and (5) the data showing archeological evidence of notable strength and muscularity in females from a diversity of small-scale populations. This work thus supports the hypothesis that higher testosterone and muscularity may have provided benefits to females inhabiting ancestral environments, as well as mediating risk of PCOS in extant populations, in conjunction with the novel environments of higher availability of food and reduced levels of exercise, and other environmental contributors, including endocrine disrupting chemicals, circadian disruption, stress, and lack of social support (9) (Figure 2B).

The benefits of higher testosterone in females, which include enhanced physical strength and athletic abilities due to anabolic effects on muscle and bone growth, and intensified competitive behavior (74, 78, 172), show evidence of applying to both lean and high-BMI females with PCOS. This finding is important because most females in ancestral populations would not have exhibited high BMI, at least not in most circumstances (173). As such, lean females with PCOS or PCOS-related traits may be relatively similar, metabolically and physiologically, to females in ancestral environments. Overall, females with PCOS (and lean females with PCOS) exhibit testosterone levels nearly twice as high, on average, compared to healthy matched controls, but with substantial overlap between the distributions, as shown in Figure 1. As such, many healthy females, and most female athletes, exhibit serum testosterone values in the range of females with PCOS; these females may exhibit subclinical PCOS traits (such as reduced ovulation rates, as described below), but also gain benefits from the advantages described above. This hypothesis could be evaluated more directly by analyzing levels of serum testosterone and muscular strength, in relation to ovulation rates and fecundability, among healthy females without PCOS.

An alternative hypothesis for the positive associations of testosterone with measures of strength and athleticism would be that exercise and strength training themselves lead to increased testosterone. Several lines of evidence contradict this hypothesis. First, exercise and strength training in females does lead to relative increases in testosterone, but the increases are transient and apparently do not lead to effects longer than several hours (174–176). Second, several studies document the strong heritability of testosterone in females, with estimates ranging from 26 to 70 percent (177–179). These findings support the idea that some females have higher testosterone levels due to genetically based factors, and not solely because of environmental factors (e.g., exercise, training, etc.). Finally, the hypothesis cannot explain the observed correlations of prenatal testosterone with strength and athleticism described above. Despite the limitations of 2D4D, this marker still provides evidence for a causal direction of the effects of prenatal testosterone on strength and athleticism in females. Overall, there is thus no clear evidence that exercise and strength training themselves lead to increases in testosterone in females.

Evidence regarding the effects of prenatal and postnatal testosterone, as well as PCOS, on BMD in females displayed notable heterogeneity across studies. A substantial proportion (about half) of the studies reported positive correlations between testosterone and BMD in healthy females; however, there were also two studies that reported negative correlations. Study results varied even more among females with PCOS compared to controls, with comparable numbers of positive, negative, and null results, such that PCOS itself cannot be considered to involve higher BMD. The differences in patterns of results between healthy females, and females with PCOS, may be attributable to factors unique to PCOS, such as insulin resistance or effects of obesity [e.g., (180)].

Systematic review also provided evidence that testosterone levels are higher in females that show higher levels of social and physical dominance, which is consistent with evidence from non-human primates and other social mammals (130, 132–135). However, dominance has yet to be studied in females with PCOS, and the links of social or physical dominance with components of fitness in human populations remain largely unexplored.

In addition to conferring potential fitness-related benefits, high testosterone in females, and in women with PCOS, also involves clear costs to reproduction. Most importantly, PCOS is a major cause of reduced fertility, as approximately 70% of females with PCOS exhibit anovulation or oligo-ovulation (181, 182). Similarly, females who have not been diagnosed with PCOS, but exhibit PCOS-associated traits, have also been shown to incur reproductive costs. Specifically, high testosterone levels and high BMI have both been linked to reduced rates of ovulation [e.g., (183–185)]. These findings are suggestive of a trade-off between investment in maintenance and survival (which is associated with higher testosterone, higher BMI, higher BMD, some degree of insulin resistance, and associated fat storage more in visceral than gluteofemoral deposits), compared to investment in higher fertility and faster reproduction (which is associated with lower testosterone) (13). Such trade-offs have yet to be investigated in females but are consistent with a model whereby PCOS and endometriosis represent diametric disorders that reflect, in part, slow vs. fast life histories (12, 13).

In a life history context, the higher BMIs of females with PCOS may also represent differential investment in visceral “survival fat” that enhances survivorship during food shortages and when subject to infectious disease risks (186–188); a slower life history is also suggested by the later ages of menarche found among average-weight females with PCOS (189–191), and the later age of menopause found among females with this condition (192–194). Among healthy females, later age of menarche has been associated with a suite of traits, including reduced mortality rates, and a longer lifespan (195–198). Such life history considerations, the hypothesized links between testosterone-related phenotypes in females, and the dysregulation of PCOS-associated traits due to modern environments, are depicted in Figure 2.

While reduced ovulation rates represent a clear correlate of high testosterone and PCOS, such reductions in fertility are reversible and contingent on physiological condition. Thus, weight loss in non-ovulating, high-BMI females commonly results in the restoration of normal ovulation (199–201). For example, Pasquali et al. (199) found that a mean weight loss of about 10 kg in 20 obese amenorrhoeic hyperandrogenic females led to the resumption of menstrual cycles in 14 (70%) of them. Similarly, Clark et al. (201) reported that after a mean weight loss of 6.5 kg, 90% of previously anovulatory obese females were ovulating spontaneously by the fifth month of the weight loss program. These findings indicate that the reproductive costs of PCOS, in terms of reduced ovulation rates, may be considerably reduced in average-weight females compared to those who are overweight. Thus, females with PCOS in ancestral populations, who were probably lean under most conditions, may have incurred relatively few reproductive costs from relatively high testosterone in terms of ovulation rates, compared to females in modern populations.

## Limitations and implications

The main limitation of this study is that although the reviews conducted are systematic, and thus unbiased regarding the hypotheses addressed, they are not exhaustive, nor have the findings been subject to meta-analyses. As such, further studies would be needed for more detailed, and functionally in-depth, analyses for any specific hypothesis of the many addressed here.

The findings reported here have several implications for future research and for treatment of PCOS in the context of its fitness-related costs and benefits. First, this work has identified a number of data gaps, including lacks of information on: (1) social and physical dominance in females with PCOS, (2) muscularity and BMD in lean females with PCOS, (3) whether female strength and muscularity are associated with markers of prenatal testosterone (especially anogenital distance); (4) levels of testosterone in females from traditional populations who engage in heavy labor, and (5) the means whereby dominant, high-testosterone females of non-human mammalian species apparently avoid the physiological, PCOS-related costs of high testosterone.

Second, if risk for PCOS evolved in the context of ecological situations favoring high levels of labor by females, and relatively high testosterone and muscularity, then treatments for PCOS may usefully focus on weight-bearing and high-intensity resistance training exercise. Such exercise, which has demonstrated positive effects on females with PCOS in the few studies conducted to date (202, 203), in part through effects in reducing insulin resistance (204), may represent the behavioral-ecological condition most relevant to the selective pressures that led to PCOS-related traits. As such, strength and resistance training should tend to simulate ancestral conditions for females with PCOS-related traits, and, in theory, may promote more-regular ovulation, insulin sensitivity, and higher fertility more effectively than other forms of exercise or treatment.

## Conclusions

This overview of systematic reviews provides evidence that the risks of PCOS in current populations may, in part, reflect extreme and maladaptive manifestations of adaptive traits. These traits, particularly those linked with higher testosterone levels, appear to offer benefits to females in terms of muscularity, athletic performance, strength, and dominance. The findings also provide support for PCOS evidence-based guidelines for physical activity, whereby treatments for PCOS could usefully focus on weight loss, strength training, and resistance training exercises.
