# Supplementary material for: The evolutionary basis of elevated testosterone in women with polycystic ovary syndrome: an overview of systematic reviews of the evidence
Source: Front Reprod Health. 2024 Sep 30;6:1475132. doi: 10.3389/frph.2024.1475132 (PMC11471738; doi:10.3389/frph.2024.1475132)
Supplement: Supplementary file 1 [file Table1.docx]

| Evidence (significant refers to p < 0.05)  **Supplementary Table 1.** Comparison of strength, musculature, and bone mineral density (BMD) among females in evidence from the archaeological record | Reference |
| --- | --- |
| Over the past 100 years, data from 63 foraging societies across the globe suggested that 50 (79%) of these groups had documentation on women hunting. Of these 50 societies, 41 societies documented whether hunting was intentional or opportunistic, where 36 (87%) of these 41 societies described women hunting as intentional | [S1] |
| Aleutian Island females (1100–1700 C.E.) had elevated humeral strength compared to Far North Coastal (500–900 C.E.), Coastal Bay and Inland/Riverine females (1500–1800 C.E.). Aleutian Island females also showed statistically similar humeral robusticity compared to Island/Riverine male hunter-gatherers | [S2] |
| Data from individuals from four burial sites in Norway dating to the Late Iron Age (750–1030 CE) and the medieval period (1030–1536 CE) were used in this study to measure BMD. Young medieval females had the highest mean BMD of all time periods, including the modern female population, and a significantly higher mean BMD than young females from the Late Iron Age | [S3] |
| Agricultural young adult skeletons (male and females) from the Georgia Coast, USA, had significantly lower values for nearly every bone geometric property measured compared to pre-agricultural young adults. These results were more pronounced among females than males and were consistent with a decline in mechanical loading associated with the transition to agriculture | [S4] |
| Ancestral remains of Far North Coastal populations (600–1500 CE) in Alaska showed significantly lower sexual dimorphism in the context of cross-sectional properties at femoral and tibial midshafts compared to other Coastal Bay and Inland populations, which was likely due to females engaging in hunting activities with males | [S5] |
| Cross sectional geometry (CSG) analysis of bones from Muisca females in Colombia showed that they had stronger and more robust humeri than males. Stronger humeri in Muisca females suggests that they were regularly engaged in strenuous agricultural activities such as grinding maize | [S6] |
| Neolithic, Bronze Age, and Iron Age female skeletal samples from ten burial sites in Central/Southeast Europe showed significantly more strengthened humeri relative to tibiae when compared to present day females and modern football players. The distribution of loading between the upper and lower limbs in early Neolithic females most closely matched the pattern documented by modern semi-elite rowers | [S7] |
| Female skeletal samples acquired across seven European regions (Britain, Scandinavia, North-Central Europe, France, Italy, the Iberian Peninsula, and the Balkans) showed a significant decrease in humeral asymmetry between the Mesolithic and Neolithic, which is correlated with new food processing techniques in the Neolithic such as two-handed grain grinding techniques | [S8] |
| Neolithic female skeletons from Poland showed significantly higher BMD values compared to early medieval, medieval, and modern times, which was likely due to a combination of diet (calcium-rich foods) and higher physical activity | [S9] |
| Neolithic female skeletons from Central Europe showed significantly higher CSG shape ratio distributions in lower limb bones compared to Iron Age females and Medieval females | [S10] |
| CSG bone analysis showed that Pottery-mound females from New Mexico, USA had robust upper bodies and strengthened humeri | [S11] |
| Alfedena (2600–2400 B.P.) female skeletons from Italy showed a significantly higher upper limb asymmetry and robusticity compared to Neolithic (6000–5500 B.P.) females, likely due to subsistence-related activities such as textile production and food processing, which may have caused a higher degree of mechanical stress on the upper limbs | [S12] |
| Late Neolithic to Early Bronze Age (5800–4000 BP) males and females from the Baikal region of Siberia showed non-significant differences in femur robusticity and a more equitable distribution of labor between males and females than Early Neolithic to Early Bronze Age populations | [S13] |
| Musculoskeletal stress markers (MSM) scores in Neolithic females from the southern Levant suggested that certain daily activities were more physically demanding than in the preceding Natufian populations. This may have been related to some of the new Neolithic activities, such as making mudbricks, preparing lime-plaster, and grinding cereals. Neolithic females also took over a greater proportion of the physical activities compared to Natufian females | [S14] |
| BMD of females and males from the medieval period (1050–1536 AD) in Norway were significantly higher than modern BMD values for males and females, but there were no significant differences in BMD when the other two time periods were compared to modern values | [S15] |
| Upper limb asymmetry significantly declined in Neolithic Ligurian females from Italy compared to European Later Upper Paleolithic females (6000–5500 B.P.), likely affected by the operation of grinding cereals and processing food | [S16] |
| CSG bone analysis showed that Arikara females from the Great Plains, USA, had a strengthened left humeri (when standardized for body size) but less humeral asymmetry compared to males. Increases in humeral CSG were associated with increased workloads among the Arikara that produced surplus crops for trade | [S17] |
| In both the Natufian and the Neolithic female populations from the Levant, some muscles exhibited higher MSM scores compared to male populations. These were mainly distal muscles, which are responsible for fine hand movements. This suggested a division of labor between the sexes, with females responsible for activities that demanded more delicate and precise movements of the hand (basketry, spinning, and weaving) | [S18] |
| Female skeletal samples from Illinois exhibited significant increases in many measures of long bone strength and external dimensions, specifically femoral and humeral strength, from the Middle Woodland to Late Woodland periods (50–1050 AD). These increases were associated with an increase in native seed crop use | [S19] |
| In Archaic hunter-gatherers and farmers from Mississippian-period Alabama (USA), maize agriculture was more physically demanding than hunting and gathering and affected females more than males. Farming led to a sexual division of labor whereby females took on a greater portion of the subsistence activities | [S20] |
| In archaic-period Indian Knoll populations, males enjoyed a reduced workload while females were exposed to an increased workload with the adoption of agriculture. In this population, farming seems to have resulted in a significant redistribution of workload by sex | [S21] |
| Femora percent cortical area (CA) was significantly higher in Atapuerca Sima de los Huesos (SH) (~430,000 BP) females in Spain than RH females in all three bone sections, and SH females showed consistently larger percent CA than SH males at all bone sections. Upper Paleolithic (UP) (50,000 to 12,000 BP) and recent population (RH) females showed significantly higher percent CA than UP and RH males all sections when standardized for body size | [S22] |
| Bone volume fraction (BV/TV), the distance between trabeculae (Tb.Sp), and degree of anisotropy (DA) values were all significantly higher in Neolithic females (~5700-4900 BP, samples collected from Germany) compared to contemporary females, likely an effect of the persistent and monotonous work for Neolithic females | [S23] |

*BMD = bone mineral density, CSG = cross-sectional geometry, MSM = musculoskeletal stress marker, CE = Common Era, BP = Before Present, AD = Anno Domini*
