# Supplementary material for: The evolutionary basis of elevated testosterone in women with polycystic ovary syndrome: an overview of systematic reviews of the evidence
Source: Front Reprod Health. 2024 Sep 30;6:1475132. doi: 10.3389/frph.2024.1475132 (PMC11471738; doi:10.3389/frph.2024.1475132)
Supplement: Supplementary file 2 [file Table2.docx]

**Supplementary Table 2.** Comparisons of serum or salivary testosterone levels between female athletes and controls using percent difference values

| Type of Athletic Activity | Form of T | Athletes mean T ± SD (N) | Controls  mean T ± SD (N) | Percent Difference | Reference |
| --- | --- | --- | --- | --- | --- |
| Russian athletes from a variety of power-based and endurance-based sports* | Serum | 1.65 ± 0.87 (599) | 1.76 ± 0.06 (298) | -6.25 | [S24] |
| Olympic athletes from a variety of power-based and endurance-based sports | Serum | 1.0 ± 0.4 (106) | 1.0 ± 0.4 (117) | 0 | [S25] |
| Elite swimmers | Serum | 2.2 ± 0.59 (25) | 1.32 ± 0.49 (21) | 66.67 | [S26] |
| Olympic athletes from a variety of power-based and endurance-based sports | Serum | 1.0 ± 0.37 (94) | 0.99 ± 0.40 (86) | 1.01 | [S27] |
| Swimmers | Serum | 1.9 ± 0.7 (18) | 1.4 ± 0.4 (18) | 35.71 | [S28] |
| Endurance athletes | Serum | 1.01 ± 0.30 (15) | 0.87 ± 0.29 (16) | 16.09 | [S29] |
| Endurance athletes | Serum | 0.60 ± 0.20 (8) | 0.70 ± 0.20 (8) | -14.28 | [S30] |
| Endurance athletes | Serum | 3.5 ± 0.50 (9) | 2.4 ± 0.6 (6) | 45.83 | [S31] |
| Olympic-level field hockey athletes | Salivary | 0.145 ± 0.054 (22) | 0.103 ± 0.054 (87) | 40.77 | [S32] |
| Elite athletes from a variety of endurance-based and power-based sports | Salivary | 0.13 ± 0.08 (6) | 0.06 ± 0.03 (16) | 116.67 | [S33] |
| Elite athletes from a variety of power-based and endurance-based sports | Salivary | 0.133 ± NA (9) | 0.07 ± NA (21) | 90.43 | [S34] |
| Elite international athletes from a variety of power-based and endurance-based sports | Salivary | 0.302 ± NA (9) | 0.142 ± NA (9) | 112.67 | [S35] |

*T = testosterone, SD = standard deviation, N = sample size*

Relevant studies that fit the search criteria were selected from Table 3. Percent difference values were calculated by using the formula (A-B/B) x 100%, where A was the mean testosterone level for women athletes and B was the mean testosterone level for controls in each respective study. All articles using Russian female athletes from the National teams tested negative for doping substances.
