# Supplementary material for: The evolutionary basis of elevated testosterone in women with polycystic ovary syndrome: an overview of systematic reviews of the evidence
Source: Front Reprod Health. 2024 Sep 30;6:1475132. doi: 10.3389/frph.2024.1475132 (PMC11471738; doi:10.3389/frph.2024.1475132)
Supplement: Supplementary file 3 [file Table3.docx]

**Supplementary Table 3.** Digit ratios (2D:4D) in relation to female athletic performance

| Participants | Findings (significant refers to p < 0.05) | Reference |
| --- | --- | --- |
| 24 elite female futsal players, mean age: 24 | Total test-time and performance time for the futsal specific performance test were significantly positively associated with dominant hand 2D:4D | [S36] |
| 532 female and 303 male undergraduate students, aged 17-21 years | Left and right 2D:4D were significantly and negatively correlated with vital capacity, 50-m run, and 800-m run in females | [S37] |
| 15 adolescent females and 11 adolescent males who were elite distance runners, mean age: 15 | Right 2D:4D was significantly negatively correlated with ventilatory threshold for females. Ventilatory threshold is a strong predictor of endurance performance | [S38] |
| 104 Swedish female Olympic athletes and 117 sedentary controls, mean age: 26 | The right 2D:4D ratio was significantly lower in female Olympic athletes compared to controls, although the left 2D:4D was not | [S39] |
| 33 female athletes and 75 sedentary females, mean age: 18 | There was no significant difference in mean 2D:4D between female athletes and sedentary females | [S40] |
| 77 college-aged female non-athletes, 103 female varsity athletes, and 78 female club sport athletes  *No ages were reported | There were no significant differences between the 2D:4Ds of athletes and non-athletes, but females who played overhand throwing sports (softball, water polo) had significantly lower 2D:4Ds than females from other sports | [S41] |
| 69 female elite rowers, mean age: 19 | Females with smaller right and left 2D:4Ds had faster race times than females with larger 2D:4Ds. Correlations were significantly positively weak to moderate for all females, moderate-to-strong for senior rowers, and weak for junior rowers | [S42] |
| 103 females and 439 males that ran a half marathon race, female mean age: 29 | Both right and left 2D:4D was significantly positively associated with half-marathon times in females | [S43] |
| 68 female collegiate tennis athletes, and 86 female nonathletes aged 18-22 years | Collegiate tennis athletes (non-elite and elite) had significantly lower right 2D:4D ratios than those of nonathletes | [S44] |
| 240 female handball athletes and 62 female controls, female mean age: 14 | Female athletes showed significantly lower right and left 2D:4Ds compared to controls | [S45] |
| 99 male and female varsity athletes, and 122 male and female student non-athletes  *No ages were reported | Female varsity athletes showed significantly lower dominant hand 2D:4Ds than non-varsity females | [S46] |
| 77 female and 70 male student rowers from the University of Cambridge, mean age: 20 in females | There were no significant associations between rowing performance levels and right/left 2D:4Ds in females | [S47] |
| 63 female and 88 male Korean Olympic-level athletes, and 88 female and 95 male non-athletic controls, mean age: 20 in females | Right and left 2D:4D was significantly lower in both elite sprint/power athletes and endurance/middle-power athletes compared to control groups in females | [S48] |
| 41 female and 58 male fencers, mean age: 24 | Lower right 2D:4D was associated with better national fencing rankings in females only, but was not significant | [S49] |
| 40 female endurance running athletes, mean age: 34 | Right 2D:4D was significantly positively related to mean finishing position | [S50] |
| 25 female rugby players, 31 female soccer players, and 26 female basketball players, mean age: 20 | Right 2D:4D showed a significant positive correlation with sports rank | [S51] |
| 607 females, mean age: 54 | Mean 2D4D (both hands) was significantly negatively associated with the highest achieved level of participation in any sport/running level | [S52] |
| 175 teenage females and 114 teenage males, female mean age: 17 | There was a significant negative correlation between right 2D:4D and standing broad jump scores for females. Physical education grade in females was significantly negatively correlated with right and left 2D:4D | [S53] |
| 24 elite female athletes and 41 non-elite female athletes (aged 20-25 years), and 73 females were in the control group (aged 18-22 years) | Elite female athletes had significantly lower left hand 2D:4D ratios than non-athletic controls | [S54] |
| 150 Iranian and 150 Afghan girls, mean age: 10 | Right left 2D:4D were significantly negatively correlated with handgrip strength and sit and reach tests, but only left 2D:4D, not right 2D:4D, was significantly negatively correlated with handgrip strength | [S55] |
| 167 female students, where 119 voluntarily chosen aerobic exercise and 48 opted for judo/boxing, mean age: 23 | Mean 2D:4D values were significantly lower in the judo/boxing athlete group compared to the aerobics group | [S56] |
| 922 boys and 835 girls, mean age: 10 | 2D:4D was not significantly correlated with scores in sprinting, shuttle run, standing broad jump and handgrip strength in girls | [S57] |
| 145 world-class female gymnasts, mean age: 16 | 2D:4D was not significantly associated with gymnastics performance level across low, middle, and high-performance groups | [S58] |
| 257 girls and 284 boys, aged 9-10 years | 2D:4D was not significantly correlated with standing long jump scores in females | [S59] |
| 64 female semi-professional basketball players, mean age: 22 | 2D:4D was significantly negatively correlated with defensive statistics in basketball games (e.g., blocks and rebounds), efficiency of scoring and was lower in females within the starting lineup compared to females on the bench | [S60] |
| 178 adolescent girls, mean age 13.5-18 | Left 2D:4D was not significantly correlated with any physical fitness components (e.g., balance, flexibility, strength, speed) | [S61] |
| 39 female and 41 male elite swimmers, mean age: 20 | Right and left 2D:4D in right-handed females was significantly negatively correlated with hand grip strength, but there were no correlations in left-handed females | [S62] |
| 24 competitive female swimmers and 34 non-athletic volunteers, mean age: 14 | Right 2D:4D, but not left 2D:4D, was significantly shorter in female competitive swimmers compared to non-athletes | [S63] |
| 30 female and 30 male basketball players, aged 15 | There were no significant correlations between 2D:4D and the Shuttle Run Test or basketball shooting efficiency in females | [S64] |
| 234 female and 359 male primary school children, aged 10-12 years | Both hands 2D:4D was significantly negatively associated with the push-up test, hand grip strength, and standing broad jump, but was not significantly associated with the Step test (measure of endurance) or 1-min abs test | [S65] |
| 48 freestyle female wrestlers, mean age: 21 | 2D:4D was not significantly associated with wrestling success in female wrestlers | [S66] |
| 36 female and 36 male rugby players and 40 female and 40 male non-athletes, aged 18-20 | 2D:4D was significantly shorter in female rugby players compared to non-athletes | [S67] |
| 36 female volleyball players, 33 female soccer players, and 34 female controls, mean age: 22 | Females engaged in contact sports had significantly shorter 2D:4D than females engaged in non-contact sports | [S68] |
| 44 female athletes and 44 controls (mean age 20), and 39 ballet dancers (mean age 29) | Left 2D:4D was significantly negatively associated with cross dominance, but positively associated with hand grip strength in female athletes | [S69] |
| 227 females and 119 males, mean age: 18 | 2D:4D was significantly negatively correlated with hand grip strength in females | [S70] |
| 59 female and 59 male swimmers, mean age: 20 | Right 2D:4D was not significantly correlated with swimming learning performance | [S71] |

*2D:4D = second-to-fourth digit ratio*
