# Supplementary material for: The evolutionary basis of elevated testosterone in women with polycystic ovary syndrome: an overview of systematic reviews of the evidence
Source: Front Reprod Health. 2024 Sep 30;6:1475132. doi: 10.3389/frph.2024.1475132 (PMC11471738; doi:10.3389/frph.2024.1475132)
Supplement: Supplementary file 4 [file Table4.docx]

**Supplementary Table 4.** Comparisons of serum testosterone levels across a sample of overweight (BMI > 25 kg/m^2^ to < 30 kg/m^2^) and obese (BMI > 30 kg/m^2^) women diagnosed with PCOS compared to controls

| PCOS BMI ± SD/[R] | PCOS mean T ± SD/[R] (N) | Control BMI ± SD/[R] | Control mean T ± SD/[R] (N) | Percent Difference | Reference |
| --- | --- | --- | --- | --- | --- |
| 28.48 ± 6.02 | 3.10 ± 1.20 (45) | 26.6 ± 5.77 | 2.60 ± 1.0 (52) | 19.23 | [S72] |
| 24.8 (23.9-25.7) | 1.79 ± 0.50 (106) | 24.1 (23.1-25.1) | 1.08 ± 0.30 (80) | 65.74 | [S73] |
| 27.6 (26.2- 29.0) | 1.46 ± (1.0-1.98) (312) | 28.3 (25.1-29.2) | 1.17 ± (0.91-1.53) (32) | 24.79 | [S74] |
| 33.9 [31.9-37.1) | 1.66 ± (1.2-2.3) (382) | 33.7 (32.0-37.4) | 1.20 ± (0.86-1.55) (131) | 38.33 | [S74] |
| 26.5 ± 6.6 | 2.9 ± 1.0 (145) | 23.7 ± 4.4 | 1.6 ± 0.40 (687) | 81.25 | [S75] |
| 25.1 ± 1.5 | 2.70 ± 0.10 (49) | 25.1 ± 1.9 | 1.50 ± 0.50 (40) | 80.0 | [S76] |
| 26.6 (20.2, 48.4) | 1.71 ± 0.97 (52) | 27.8 (21.1, 33.3) | 0.84 ± NA (42) | 103.57 | [S77] |
| 25.0 (19-51) | 2.10 ± NA (69) | 21.68 (18.24-50.15) | 1.10 ± NA (41) | 90.91 | [S78] |
| 34.55 ± 5.66 | 3.70 ± 1.09 (33) | 34.16 ± 8.05 | 1.19 ± 0.48 (49) | 210.9 | [S79] |
| 30.1 ± 2.8 | 2.8 ± 1.20 (39) | 28.5 ± 1.6 | 1.8 ± 0.30 (30) | 55.56 | [S80] |
| 27.5 ± 5.8 | 2.50 ± 1.14 (95) | 26.8 ± 3 | 1.21 ± 0.45 (90) | 106.61 | [S81] |
| 27.5 ± 6.5 | 1.80 ± 0.50 (29) | 23.7 ± 6.7 | 1.50 ± 0.50 (22) | 20 | [S82] |
| 31.3 ± 8.7 | 2.40 ± 0.90 (60) | 30.7 ± 7.5 | 1.35 ± 0.52 (34) | 77.78 | [S83] |
| 27.6 ± 1.8 | 2.03 ± 0.89 (16) | 26.8 ± 2.8 | 0.82 ± 0.43 (16) | 147.56 | [S84] |
| 35.8 ± 3.5 | 2.52 ± 1.82 (15) | 33.7 ± 3.7 | 0.96 ± 0.51 (15) | 162.5 | [S84] |
| 31.3 ± 8.45 | 1.83 ± 1.28 (84) | 28.9 ± 7.63 | 1.05 ± 0.64 (37) | 74.29 | [S85] |
| 31.6 (25.1-47.8) | 2.80 ± (1.1-6.9) (54) | 21.9 (18.6-24.5) | 1.70 ± (0.9-2.3) (19) | 64.71 | [S86] |

*PCOS = polycystic ovary syndrome, T = testosterone, R = range, N = sample size, BMI = body mass index*

Percent difference values were calculated by using the formula (A-B/B) x 100%, where A was the mean testosterone level for the overweight/obese women with PCOS and B was the mean testosterone level for the controls in each respective study. Due to the significantly large number of articles returned from the Web of Science database search, a random sample of 17 studies from the search records that fit the inclusion criteria were analysed.
