# Supplementary material for: The evolutionary basis of elevated testosterone in women with polycystic ovary syndrome: an overview of systematic reviews of the evidence
Source: Front Reprod Health. 2024 Sep 30;6:1475132. doi: 10.3389/frph.2024.1475132 (PMC11471738; doi:10.3389/frph.2024.1475132)
Supplement: Supplementary file 5 [file Table5.docx]

**Supplementary Table 5.** Comparisons of serum testosterone levels across a sample of lean

(BMI < 25 kg/m^2^) women diagnosed with PCOS and controls

| PCOS BMI ± SD/(R) | Lean PCOS mean T ± SD/(R)(N) | Control BMI ± SD/(R) | Control mean T ± SD/(R) (N) | Percent Difference | Reference |
| --- | --- | --- | --- | --- | --- |
| 21.5 (22.0-30.0) | 1.50 ± (1.07-2.09) (350) | 21.0 (20.0-22.2) | 1.14 ± (0.9-1.44) (203) | 31.58 | [S74] |
| 21.37 ±1.90 | 3.44 ± 1.29 (38) | 21.46 ± 1.67 | 1.24 ± 0.47 (51) | 177.42 | [S79] |
| 22.0 ± 1.6 | 2.8 ± 1.30 (44) | 21.4 ± 1.6 | 1.9 ± 0.40 (34) | 47.37 | [S80] |
| 19.4 ± 1.6 | 1.0 ± 0.50 (6) | 19.6 ± 1.6 | 0.70 ± 0.20 (8) | 42.86 | [S30] |
| 22.2 ± 2.5 | 1.84 ± 1.55 (15) | 23.4 ± 2.9 | 0.85 ± 0.42 (15) | 116.47 | [S84] |
| 21.4 (16.8-24.5) | 2.50 ± (1.3-6.0) (48) | 21.9 (18.6-24.5) | 1.70 ± (0.9-2.3) (19) | 47.06 | [S86] |
| 22.22 ± 1.64 | 1.04 ± (0.66-1.56) (91) | 21.97 ± 1.45 | 0.52 ± (0.31-0.79) (45) | 100.00 | [S87] |
| 20.78 ± 2.23 | 1.35 ± 0.66 (17) | 20.11 ± 1.91 | 0.94 ± 0.42 (17) | 43.62 | [S88] |
| 22.4 ± 3.04 | 1.65 ± 0.28 (30) | 23.1 ± 3.06 | 1.15 ± 0.21 (17) | 43.48 | [S89] |
| 24.66 ± 0.38 | 1.56 ± 0.07 (50) | 24.05 ± 0.55 | 1.08 ± 0.04 (40) | 44.44 | [S90] |
| 23.5 ± 0.4 | 2.88 ± 0.66 (4) | 22.4 ± 0.5 | 1.18 ± 0.26 (4) | 122.09 | [S91] |
| 21.9 (20.2-23.5) | 3.2 ± (2.8-3.6) (8) | 20.1 (18.9-21.2) | 2.3 ± (0-3.3) (7) | 39.13 | [S92] |

*PCOS = polycystic ovary syndrome, T = testosterone, BMI = body mass index, SD = standard deviation, R = range, N = sample size*

Due to the large number of relevant articles returned from the Web of Science (WOS) database search, only the first 6 studies from the WOS search, sorted by relevance, the 5 relevant studies that included a population of lean women with PCOS and controls in Supplementary Table 4, and the 1 relevant study in Supplementary Table 2 were all included in this analysis. Percent difference values were calculated by using the formula (A-B/B) x 100%, where A was the mean testosterone level for the lean women with PCOS and B was the mean testosterone level for the controls in each respective study.
