# Supplementary material for: The evolutionary basis of elevated testosterone in women with polycystic ovary syndrome: an overview of systematic reviews of the evidence
Source: Front Reprod Health. 2024 Sep 30;6:1475132. doi: 10.3389/frph.2024.1475132 (PMC11471738; doi:10.3389/frph.2024.1475132)
Supplement: Supplementary file 6 [file Table6.docx]

**Supplementary Table 6.** Comparisons of serum testosterone levels across samples of lean women with PCOS (BMI < 25 kg/m^2^), non-lean women with PCOS, (BMI > 25 kg/m^2^), and control women

| Population | Method of T Quantification | PCOS T Levels (nmol/L) | Lean PCOS T Levels (nmol/L) | Control T Levels (nmol/L) | Reference |
| --- | --- | --- | --- | --- | --- |
| 45 women with PCOS and 52 control women aged 18-37 years of age | RI | 3.1 ± 1.2  (P < 0.05) | N/A | 2.6 ± 1.0 | [S72] |
| 106 women with PCOS (mean = 26 years) and 80 control women (mean = 28 years) | CS | 1.79 ± 0.5  (P < 0.001) | N/A | 1.08 ± 0.3 | [S73] |
| 1044 women with PCOS and variable BMIs and 366 control women without PCOS. Mean age = 26 | RI | 1.6 (1.2, 2.3) obese and overweight  (P < 0.05) | 1.5 (1.1, 2.1)  (P < 0.05) | 1.1 (0.9, 1.4) lean controls, 1.2 (0.9, 1.5) obese and overweight | [S74] |
| 145 women with PCOS and 687 controls. All subjects were 31 years of age | CS | 2.9 ± 1.0  (P = 1.2 x10^-34^) | N/A | 1.6 ± 0.4 | [S77] |
| 52 women with PCOS aged 18-35 years and 42 aged-matched control subjects | LC-MS/MS | 1.71 ± 0.97  (P < 0.001) | N/A | 0.84 | [S77] |
| 69 women with PCOS and 41 control group women aged between 18 and 43 years | CS | 2.10  (P < 0.001) | N/A | 1.10 | [S78] |
| 38 lean PCOS women (mean = 29 years), 51 lean controls (mean = 31 years), 33 obese PCOS (mean = 27 years), and 49 obese controls (mean = 33 years) | EI | 3.70 ± 1.09  (P < 0.0001) | 3.44 ± 1.29  (P < 0.0001) | 1.24 ± 0.47 lean controls, 1.19 ± 0.48 obese | [S79] |
| 95 women with PCOS and 90 age and weight-matched controls  Between 18 and 40 years (mean age 24 years) | RI | 2.50 ± 1.14  (P < 0.05) | N/A | 1.21 ± 0.45 | [S81] |
| 44 lean PCOS women (mean = 23 years), 34 lean controls (mean = 24 years), 39 overweight PCOS women (mean = 26 years), and 30 overweight controls (mean = 28 years) | EI | 2.8 ± 1.2  (P < 0.001) | 2.8 ± 1.3  (P < 0.001) | 1.9 ± 0.4 lean controls, 1.8 ± 0.3 overweight | [S80] |
| 29 women with PCOS (mean = 27 years) and 22 control women (mean = 26 years) | CS | 1.8 ± 0.5  (P = 0.07) | N/A | 1.5 ± 0.5 | [S82] |
| 6 women athletes in endurance sports with PCOS aged 16-35 years, and 8 inactive women in in the control group | RI | N/A | 1.0 ± 0.5  (P < 0.05) | 0.7 ± 0.2 | [S30] |
| 60 women with PCOS (mean = 25 years) and 34 control women (mean = 31 years) | RI | 2.40 ± 0.90  (P < 0.001) | N/A | 1.35 ± 0.52 | [S83] |
| 46 women with PCOS and 46 control women age/weight matched (mean = 39 years) | RI | 2.03 ± 0.89 overweight (n = 16), 2.52 ± 1.82 obese (n = 15)  (P < 0.05) | 1.84 ± 1.55  (n = 15)  (P < 0.01) | 0.85 ± 0.42 lean controls, 0.82 ± 0.43 overweight, and 0.96 ± 0.51 obese | [S84] |
| 84 women with PCOS and 37 controls with a mean age of 41 years | RI | 1.83 ± 1.28  (P < 0.001) | N/A | 1.05 ± 0.64 | [S85] |
| 48 lean women with PCOS, 54 obese women with PCOS, and 19 lean controls (no ages reported) | EI | 2.8 (1.1-6.9)  (P < 0.01) | 2.5 (1.3-6.0)  (P < 0.01) | 1.7 (0.9-2.3)  (P < 0.01) | [S86] |
| 91 lean women with PCOS and 45 lean controls (mean = 30 years) | N/A | N/A | 1.04 (0.66-1.56)  (P < 0.05) | 0.52 (0.31-0.79) | [S87] |
| 30 lean women with PCOS and 17 lean controls (mean = 26 years) | EI | N/A | 1.65 ± 0.28  (P < 0.02) | 1.15 ± 0.21 | [S89] |
| 4 lean women with PCOS (mean = 21 years) and 4 lean controls (mean = 28 years) | RI | N/A | 3.2 (2.8-3.6)  (P < 0.05) | 2.3 ± (0-3.3) | [S92] |

*PCOS = polycystic ovary syndrome, T = testosterone, BMI = body mass index, LC-MS/MS = liquid chromatography tandem mass spectrometry, RI = radioimmunoassay, EI = enzyme immunoassay, CS = chemiluminescence assay system*

Previously selected studies of lean and overweight women from Supplementary Table 4 (n=14) and Supplementary Table 5 (n=4) were selected for analysis. The p-value was calculated based on the comparison of each group (overweight/obese/lean PCOS women) with its respective weight-matched control group.
