# Supplementary material for: The evolutionary basis of elevated testosterone in women with polycystic ovary syndrome: an overview of systematic reviews of the evidence
Source: Front Reprod Health. 2024 Sep 30;6:1475132. doi: 10.3389/frph.2024.1475132 (PMC11471738; doi:10.3389/frph.2024.1475132)
Supplement: Supplementary file 7 [file Table7.docx]

| Participants  **Supplementary Table 7.** Comparison of bone mineral density (BMD) values in females with PCOS vs controls | Findings (significant refers to p < 0.05) | Reference |
| --- | --- | --- |
| 158 pre-menopausal females with PCOS and 25 age- and BMI-matched controls  *No ages were reported | Females with PCOS showed significantly lower BMD in the L1-L4 vertebrae, independent of BMI. The femoral neck, the trochanter region, and total skeleton BMD were not significantly different from controls | [S93] |
| 55 females with PCOS (mean age 25) and 60 controls (mean age 30) | Females with PCOS showed significantly lower total BMD, upper limb BMD and lower limb BMD compared to controls | [S94] |
| 123 females with PCOS (mean age 16) and 47 controls (mean age 17) | There was no significant difference in BMD z-score between females with PCOS and controls | [S95] |
| 60 females with PCOS and 58 controls aged 14-24 years | There was no significant difference in mean BMD between females with PCOS and controls | [S96] |
| 60 females with PCOS (mean age 26) and 60 controls (mean age 27) | BMI-corrected whole-body BMD as well as the lumbar spine and regions of proximal femur were not significantly different between females with PCOS and controls | [S97] |
| 103 females with PCOS (mean age 26) and 60 BMI-matched controls (mean age 27) | Lumbar BMD and femoral neck BMD were significantly lower in the PCOS group compared to the control group | [S98] |
| 69 reproductive-aged females with PCOS (mean age 23) and 30 controls (mean age 24) | Females with PCOS showed significantly lower BMD values compared to controls | [S99] |
| 37 females with PCOS aged 16-18 and 40 normal weight eumenorrheic girls | Females with PCOS showed significantly lower lumbar spine BMD values compared to controls | [S100] |
| 30 females with classic PCOS (mean age 23), 13 with ovulatory PCOS (mean age 22), and 22 controls (mean age 27) | There were no significant differences in lumbar spine and femur BMD between in comparison to the control group | [S101] |
| 25 postmenopausal females with PCOS aged 61-78 and 68 randomly allocated age-matched controls | There was no significant difference in mean BMD in females with PCOS compared to controls | [S102] |
| 30 females with PCOS (15 lean and 15 obese with mean ages of 26 and 28, respectively) and 15 controls (mean age 26) | Volumetric cortical density BMD was significantly higher in females with PCOS compared to controls and furthermore in lean females with PCOS compared to obese ones | [S103] |
| 41 females with hypothalamic amenorrhea (HA) + PCOS (mean age 23), and 41 females with HA as a comparison group (mean age 24) | The HA + PCOS group showed significantly higher hip and spine bone densities compared to the HA group | [S104] |
| 29 patients with PCOS (mean age 28) and 17 BMI-matched controls (mean age 29) | There were no significant differences in total body, lumbar spine, and femoral neck BMD between females with PCOS and controls | [S105] |
| 10 lean females with PCOS (mean age 23) and 10 lean controls (mean age 24) | Lean females with PCOS showed a non-significant difference in bone density compared to lean controls | [S106] |
| 28 amenorrheic females with PCOS (mean age 23), 11 amenorrheic females without PCOS (mean age 22), and 15 controls (mean age 23) | BMD in the PCOS group was significantly lower than in the control group | [S107] |
| 12 females with PCOS (mean age 28) and 10 ethnicity and weight-matched controls (mean age 29) | Lean females with PCOS showed significantly increased BMD in the upper skeleton compared with controls | [S108] |
| 51 females with PCOS (mean age 24) and 35 controls (mean age 26) | There were no significant differences in spine and femoral BMD between females with PCOS and controls | [S109] |
| 80 females with PCOS and 15 controls (mean age 28) | BMD of the spine and femur were significantly higher in the PCOS group compared to the control group | [S110] |
| 52 females with PCOS (mean age 22) and 39 controls (mean age 26) | There were no significant differences in BMD between females with PCOS and controls | [S111] |
| 59 females with phenotypes A and B, PCOS (mean age 26), 23 females with type C PCOS (mean age 29), and 51 controls (mean age 28) | Females with types A, B, and C PCOS had significantly higher bone mass, lumbar spine and total femur BMD than in controls | [S112] |
| 21females with PCOS (mean age 40) and 39 controls (mean age 42) | There were no significant differences in measures of BMD between females with PCOS and controls | [S113] |

*PCOS = polycystic ovary syndrome, BMD = bone mineral density, BMI = body mass index*

Due to the large quantity of studies returned on Google Scholar, only the first 5 relevant studies, sorted by relevance, that fit the Google Scholar search criteria were analysed.
