# Supplementary material for: The evolutionary basis of elevated testosterone in women with polycystic ovary syndrome: an overview of systematic reviews of the evidence
Source: Front Reprod Health. 2024 Sep 30;6:1475132. doi: 10.3389/frph.2024.1475132 (PMC11471738; doi:10.3389/frph.2024.1475132)
Supplement: Supplementary file 8 [file Table8.docx]

**Supplementary Table 8.** Serum and salivary testosterone levels in relation to measures of bone mineral density (BMD) in healthy females

| Participants | Findings (significant refers to p < 0.05) | Reference |
| --- | --- | --- |
| 68 postmenopausal females aged 65-89 | Serum T levels were significantly positively associated with BMD t-score of the hip, but not that of the lumbar spine | [S114] |
| 111 premenopausal females, mean age 24 years | Serum T levels were not significantly associated with BMD at the lumbar spine | [S115] |
| 2198 females aged 40-60 years, (mean age 49) | Serum total T levels were significantly positively correlated with lumbar BMD | [S116] |
| 80 postmenopausal women with normal BMD (mean age 53) and 109 postmenopausal women with low BMD (mean age 57) | Participants with higher BMD at the lumbar spine and femur showed significantly higher T levels than participants with lower BMD | [S117] |
| 262 non-osteoarthritis (OA) females with a (mean age 55), 52 early knee OA (mean age 58), 204 radiographic knee OA (mean age 67) | Serum T levels were significantly and weakly positively correlated with BMD of the distal radius across all women | [S118] |
| 64 postmenopausal females, mean age 56 years | Serum T was not significantly correlated with BMDs of the lumbar spine, total femur, femoral neck, trochanteric, intertrochanteric or Ward’s triangle | [S119] |
| 25 female swimmers and 21 control subjects (mean age 15 years) | Significant but low negative correlations were observed between serum total T and areal BMD at the femoral neck and intertrochanter in female swimmers | [S77] |
| 102 females, mean age 18 years | Baseline T was significantly positively associated with cortical volumetric BMD at early adulthood, after a 7-year follow-up study | [S120] |
| 68 healthy obese premenopausal women, mean age 36 years | Serum T was not significantly associated with trabecular BMD of the lumbar spine | [S121] |
| 244 postmenopausal females, mean age 54 years | Serum T levels were significantly and positively correlated with BMD of the femur, while correlations with neck BMD and lumbar BMD were not significant | [S122] |
| 232 postmenopausal females, mean age 75 | Total T levels were significantly positively associated with BMD at the lumbar spine and hip | [S123] |
| 43 female elite rhythmic gymnasts aged 10 to 17 years | Serum T levels were significantly positively correlated with whole body BMD, L2-L4 vertebrae BMD, total proximal femur BMD, mid-radius BMD, and skull BMD | [S124] |
| 192 premenopausal females aged 40-45 years | Total serum T was significantly negatively associated with head BMD, but not with BMD of the lumbar spine and pelvis | [S125] |
| 2311 pre- and perimenopausal females aged 42-52 years at baseline | There was no significant association of lumbar spine, total hip, or femoral neck BMD change with change in levels of T | [S126] |
| 99 postmenopausal females aged 55-75 years | Serum T levels significantly and positively predicted BMD of the lumbar spine, BMD of the total femur and the total skeleton | [S127] |
| 50 premenarcheal females, mean age 12 years | Serum T levels were significantly positively related to ultradistal/proximal BMD | [S128] |
| 118 early postmenopausal women, mean age 54 years | There was no significant association between BMD and total T | [S129] |
| 60 premenopausal females with Turner Syndrome and 59 control women aged 36 years | Serum T levels were significantly and positively correlated with spine BMD and arm BMD in healthy controls | [S130] |
| 176 postmenopausal females, mean age 69 | There was no significant association between total T and total hip BMD | [S131] |
| 39 healthy or osteoporotic premenopausal women (mean age 40), and 108 postmenopausal women (mean age 62) | Serum total T levels significantly positively correlated with BMD at the spine | [S132] |
| 66 premenopausal females aged 42 years | Salivary T levels were significantly positively associated with lumbar spine BMD | [S133] |
| 457 postmenopausal females, mean age 72 years | There was no significant association between T levels and BMD | [S134] |
| 48 premenopausal females (mean age 36) and 44 postmenopausal females (mean age 70) | Serum T levels were not significantly correlated with femur or lumbar spine BMD in either group | [S135] |
| 90 postmenopausal females, mean age 58 years | There were no significant correlations between serum total T and measurements of BMD | [S136] |
| 27 female non-smokers and 25 smokers, mean age 29 | Serum T levels were not significantly related to BMDs of the whole body, lumbar spine, femoral neck, trochanter, or Ward’s triangle | [S137] |
| 93 postmenopausal females aged 74 years | Serum T levels were significantly positively correlated with radial mineral density (RMD) | [S138] |
| 20 hirsute females and 19 control females, mean age 30 years | BMD in the lumbar spine and proximal femur were not significantly correlated with serum T in the healthy control group | [S139] |
| 63 healthy controls (mean age 35) and 51 patients with hirsutism (mean age 33) | No significant correlations were found between hip and neck BMD and serum T levels in healthy controls | [S140] |
| 884 postmenopausal females (mean age 52 years) | Serum T was significantly positively correlated with neck BMD, but not lumbar BMD | [S141] |
| 91 girls (mean age 13) and 83 boys (mean age 14) | Serum T was not significantly associated with lumbar BMD in girls | [S142] |
| 248 girls (mean age 11 years) | Serum T was not significantly associated with total BMD in girls | [S143] |

*T = testosterone, BMD = bone mineral density*
