# Supplementary material for: The evolutionary basis of elevated testosterone in women with polycystic ovary syndrome: an overview of systematic reviews of the evidence
Source: Front Reprod Health. 2024 Sep 30;6:1475132. doi: 10.3389/frph.2024.1475132 (PMC11471738; doi:10.3389/frph.2024.1475132)
Supplement: Supplementary file 9 [file Table9.docx]

**Supplementary Table 9.** Digit ratios in relation to bone mineral density in healthy females

| Participants | Findings (significant refers to p < 0.05) | References |
| --- | --- | --- |
| 74 females and 102 males over 18 years of age | 2D:4D was not significantly associated with total BMD in females | [S144] |
| 127 females, mean age 31 years | Right 2D:4D was significantly and positively associated with BMD of the lumbar spine and femoral neck | [S145] |
| 97 postmenopausal females, mean age 50 years | Right 2D:4D was significantly and positively associated with BMD of the lumbar spine and the hip | [S146] |

*2D:4D = second-to-fourth digit ratio, BMD = bone mineral density*

** One study showed significant positive correlations between anogenital index (AGI), which is AGD divided by BMI, and BMD [S147]. However, this study was not included in the analysis due to previously reported strong and positive links between BMI and BMD (e.g., [S148] and [S149]), thus confounding AGD with BMD
