# Supplementary material for: The evolutionary basis of elevated testosterone in women with polycystic ovary syndrome: an overview of systematic reviews of the evidence
Source: Front Reprod Health. 2024 Sep 30;6:1475132. doi: 10.3389/frph.2024.1475132 (PMC11471738; doi:10.3389/frph.2024.1475132)
Supplement: Supplementary file 10 [file Table10.docx]

**Supplemental References**

S1. Anderson A, Chilczuk S, Nelson K *et al.* The myth of man the hunter: women’s contribution to the hunt across ethnographic contexts. *PLoS One* 2023; **18**:e0287101.

S2. Temple DH, Rosa E, Hunt DR, *et al*. Adapting in the Arctic II: upper limb diaphyseal robusticity and habitual activity in Late Holocene hunter-gatherers from Alaska. *Am J Biol Anthropol* 2023; **181**:392–412.

# S3. Brødholt ET, Günther CC, Gautvik KM, *et al*. Bone mineral density through history: dual-energy X-ray absorptiometry in archaeological populations of Norway. *J Archaeol Sci* 2021; 36:102792.

S4. Kralick A, Zemel B. Evolutionary perspectives on the developing skeleton and implications for lifelong health. *Front Endocrinol* 2020; **11**:99.

S5. Temple DH, Rosa ER, Hunt DR, *et al*. Adapting in the Arctic: habitual activity and landscape interaction in Late Holocene hunter-gatherers from Alaska. *Am J Phys Anthropol* 2021; **176**:3–20.

S6. Miller M, Agarwal S, Aristizabal L *et al.* The daily grind: sex- and age-related activity patterns inferred from cross-sectional geometry of long bones in a pre-Columbian muisca population from Tibanica, Colombia. *Am J Phys Anthropol* 2018; **167**:311–26.

S7. Macintosh A, Pinhasi R, Stock J. Prehistoric women’s manual labor exceeded that of athletes through the first 5500 years of farming in Central Europe. *Sci Adv* 2017; **3**:eaao3893.

S8. Sládek V, Ruff CB, Berner M, *et al*. The impact of subsistence changes on humeral bilateral asymmetry in Terminal Pleistocene and Holocene Europe. *J Hum Evol* 2016; **92**:37–49.

S9. Spinek AE, Lorkiewicz W, Mietlinska J *et al.* Evaluation of chronological changes in bone fractures and age-related bone loss: a test case from Poland. *J Archaeol Sci* 2016; **72**:117–27.

S10. Macintosh AA, Pinhasi R, Stock JT. Divergence in male and female manipulative behaviors with the intensification of metallurgy in Central Europe. *PLoS One* 2014; **9**:e112116.

S11. Ogilvie M, Hilton E. Cross-sectional geometry in the humeri of foragers and farmers from the prehispanic American Southwest: exploring patterns in the sexual division of labor. *Am J Phys Anthropol* 2011; **144**:11–21.

S12. Sparacello VS, Pearson OM, Coppa A, *et al*. Changes in skeletal robusticity in an iron age agropastoral group: the Samnites from the Alfedena necropolis (Abruzzo, Central Italy). *Am J Phys Anthropol* 2011; **144**:119–30.

S13. Weber AW, Bettinger R. Middle Holocene hunter-gatherers of Cis-Baikal, Siberia: an overview for the new century. *J Anthropol Archaeol* 2010; **29**:491–506.

S14. Hershkovitz I, Gopher A. Demographic, biological and cultural aspects of the Neolithic revolution: a view from the Southern Levant. In: Bocquet-Appel J-P, Bar-Yosef O (eds). *The Neolithic Demographic Transition and its Consequences*. Springer, 2008, 441–79.

S15. Holck P. Bone mineral densities in the prehistoric, Viking-Age, and medieval populations of Norway. *Int J Osteoarchaeol* 2007; **17**:199–206.

S16. Marchi D, Sparacello VS, Holt BM *et al.* Biomechanical approach to the reconstruction of activity patterns in Neolithic western Liguria, Italy. *Am J Phys Anthropol* 2006; **131**:447–55.

S17. Wescott D, Cunningham D. Temporal changes in Arikara humeral and femoral cross-sectional geometry associated with horticultural intensification. *J Archaeol Sci* 2006; **33**:1022–36.

S18. Eshed V, Gopher A, Galili E *et al.* Musculoskeletal stress markers in Natufian hunter-gatherers and Neolithic farmers in the Levant: the upper limb. *Am J Phys Anthropol* 2004; **123**:303–15.

S19. Bridges PS, Blitz JH, Solano MC. Changes in long bone diaphyseal strength with horticultural intensification in west-central Illinois. *Am J Phys Anthropol* 2000; **112**:217–38.

S20. Bridges, P. [Changes in activities with the shift to agriculture in the southeastern United States](https://www.journals.uchicago.edu/doi/abs/10.1086/203756). *Curr Anthropol* 1989; **30**:385–94.

S21. Cassidy CM. Skeletal evidence for prehistoric subsistence adaptation in the Central Ohio River Valley. In: Cohen MH (ed). *Paleopathology at the Origins of Agriculture*. Academic Press, 1984, 307–38.

S22. Carretero JM, Rodríguez L, García-González R, *et al*. Exploring bone volume and skeletal weight in the Middle Pleistocene humans from the Sima de los Huesos site (Sierra de Atapuerca, Spain). *J Anat*. 2018; **233**(6):740-754.

S23. Scherf H, Wahl J, Hublin JJ, Harvati K. Patterns of activity adaptation in humeral trabecular bone in Neolithic humans and present-day people. *Am J Phys Anthropol*. 2016; **159**(1):106-15.

S24. Ahmetov II, Stepanova AA, Biktagirova EM *et al.* Is testosterone responsible for athletic success in female athletes? *J Sports Med Phys Fitness* 2020; **60**:1377–82.

S25. Eklund E, Berglund B, Labrie F *et al.* Serum androgen profile and physical performance in women Olympic athletes. *Br J Sports Med* 2017; **0**:1–9.

S26. Maïmoun L, Coste O, Philibert P *et al.* Testosterone secretion in elite adolescent swimmers does not modify bone mass acquisition: a 1-year follow-up study. *Fertil Steril* 2013; **99**:270–8.

S27. Eklund E, Andersson A, Ekström L *et al.* Urinary steroid profile in elite female athletes in relation to serum androgens and in comparison with untrained controls. *Front Physiol* 2021; **12**:702305.

S28. Coste O, Paris F, Galtier F *et al.* Polycystic ovary-like syndrome in adolescent competitive swimmers. *Fertil Steril* 2011; **96**:1037–42.

S29. Russell M, Stark J, Nayak S, *et al*. Peptide YY in adolescent athletes with amenorrhea, eumenorrheic athletes and non-athletic controls. *Bone* 2009; **45**:104–9.

S30. Rickenlund A, Thorén M, Carlström K *et al.* Diurnal profiles of testosterone and pituitary hormones suggest different mechanisms for menstrual disturbances in endurance athletes. *J Clin Endocrinol Metab* 2004; **89**:702–7.

S31. Uusitalo AL, Huttunen P, Hanin Y *et al.* Hormonal responses to endurance training and overtraining in female athletes. *Clin J Sport Med* 1998; **8**:178–86.

S32. Casto KV, Arthur LC, Edwards DA, *et al.* Testosterone, athletic context, oral contraceptive use, and competitive persistence in women. *Adapt Hum Behav Physiol* 2022; **8**:52–78.

S33. Cook CJ, Kilduff LP, Crewther BT. Basal and stress-induced salivary testosterone variation across the menstrual cycle and linkage to motivation and muscle power. *Scand J Med Sci Sports* 2018; **28**:1345–53.

S34. Crewther BT, Cook CJ. A longitudinal analysis of salivary testosterone concentrations and competitiveness in elite and non-elite women athletes. *Physiol Behav* 2018; **188**:157–61.

S35. Cook CJ, Crewther BT, Smith AA. Comparison of baseline free testosterone and cortisol concentrations between elite and non-elite female athletes. *Am J Hum Biol* 2012; **24**:856–8.

S36. Farhani F, Arazi H, Mirzaei M, *et al*. Associations between bio-motor ability, endocrine markers and hand-specific anthropometrics in elite female futsal players: a pilot study. *BMC Sports Sci Med Rehabil* 2022; **14**:59.

S37. Chen T, Xiang J, Teng S, *et al*. Relationship between digit ratio of 2D:4D and the physical health among college students of Han ethnicity in southern Fujian. *J Anat Soc India* 2022; **71**:214–19.

S38. Lombardo MP, Otieno S. The associations between digit ratio, aerobic fitness, physical skills, and overall physical fitness of elite youth distance runners. *Am J Hum Biol* 2021; **33**:e23448.

S39. Eklund E, Ekström L, Thörngren JO *et al.* Digit ratio (2D:4D) and physical performance in female Olympic athletes. *Front Endocrinol (Lausanne)* 2020; **11**:292.

S40. Aminu AM, Udeze CN, Samaila H, *et al.* There is no sex-, handedness-, and hand grip strength-related dimorphism in digit ratio in both sedentary and athletic African young people. *J Res Med Dent Sci* 2018; **6**:108–13.

S41. Lombardo MP, Otieno S, Heiss A. College-aged women in the United States that play overhand throwing sports have masculine digit ratios. *PLoS One* 2018; **13**:e0203685.

S42. Hull MJ, Schranz NK, Manning JT *et al.* Relationships between digit ratio (2D:4D) and female competitive rowing performance. *Am J Hum Biol* 2015; **27**:157–63.

S43. Longman D, Stock JT, Wells JCK. Digit ratio (2D:4D) and rowing ergometer performance in males and females. *Am J Phys Anthropol* 2011; **144**:337–41.

S44. Hsu CC, Su B, Kan NW, *et al*. Elite collegiate tennis athletes have lower 2D:4D ratios than those of nonathlete controls. *J Strength Cond Res* 2015; **29**:822–5.

S45. Baker J, Kungl AM, Pabst J, *et al.* Your fate is in your hands? Handedness, digit ratio (2D:4D), and selection to a national talent development system. *Laterality* 2013; **18**:710–18.

S46. Giffin NA, Kennedy RM, Jones ME, *et al*. Varsity athletes have lower 2D:4D ratios than other university students. *J Sports Sci* 2012; **30**:135–8.

S47. Longman D, Stock JT, Wells JC. Digit ratio (2D:4D) and rowing ergometer performance in males and females. *Am J Phys Anthropol* 2011; **144**:337–41.

S48. Kim W, Cho HI, Kim KC *et al.* Relationships between digit ratio (2D:4D), *ACE* gene polymorphism, and physical performance in the Korean population. *Genes Genom* 2011; **33**; 407–12.

S49. Voracek M, Reimer B, Dressler SG. Digit ratio (2D:4D) predicts sporting success among female fencers independent from physical, experience, and personality factors. *Scand J Med Sci Sports* 2010; **20**:853–60.

S50. Manning JT, Morris L, Caswell N. Endurance running and digit ratio (2D:4D): implications for fetal testosterone effects on running speed and vascular health. *Am J Hum Biol* 2007; **19**:416–21.

S51. Keith-Barnett N, Campbell A. Sporting achievement: what is the contribution of digit ratio? *J Personal* 2007; **75**:663–78.

S52. Paul SN, Kato BS, Hunkin JL *et al.* The big finger: the second to fourth digit ratio is a predictor of sporting ability in women. *Br J Sports Med* 2006; **40**:981–3.

S53. Hönekopp JT, Manning J, Müller C. Digit ratio (2D:4D) and physical fitness in males and females: evidence for effects of prenatal androgens on sexually selected traits. *Horm Behav* 2006; **49**:545–9.

S54. Pokrywka L, Rachoń D, Suchecka-Rachoń K *et al.* The second to fourth digit ratio in elite and non-elite female athletes. *Am J Hum Biol* 2005; **17**:796–800.

S55. Agha-Alinejad H, Farzad B, Akbari M, *et al*. Digit ratios and motor and health-related fitness in pre-adolescent females. *Ann Hum Biol*. 2019; **46**(3):225-230.

S56. Koziel S, Kociuba Mail M, Chakraborty R, *et al*. Physical Fitness And Digit Ratio (2D:4D) In Male Students From Wrocław, Poland. *Coll Antropol*. 2017; **41**(1):31-7.

S57. Ranson R, Stratton G, Taylor SR. Digit ratio (2D:4D) and physical fitness (Eurofit test battery) in school children. *Early Hum Dev*. 2015; **91**(5):327-31.

S58. Peeters MW, Claessens AL. Digit ratio (2D:4D) and competition level in world-class female gymnasts. *J Sports Sci*. 2013;**31**(12):1302-11.

S59. Hsu CC, Fong TH, Chang HM, *et al*. Low Second-To-Fourth Digit Ratio Has High Explosive Power? A Prepubertal Study. *J Strength Cond Res*. 2018; **32**(7):2091-2095.

S60. Dyer M, Short SE, Short M, *et al*. Relationships between the second to fourth digit ratio (2D:4D) and game-related statistics in semi-professional female basketball players. *Am J Hum Biol*. 2018; **30**(1).

S61. Peeters MW, Van Aken K, Claessens AL. The left hand second to fourth digit ratio (2D:4D) is not related to any physical fitness component in adolescent girls. *PLoS One*. 2013;**8**(4):e59766.

S62. Acar H, Eler, N. The Relationship of Digit Ratio (2D:4D) With Cerebral Lateralization and Grip Strength in Elite Swimmers. *Journal of Education and Training Studies,* 2018, **6**(4):84.

S63. Günay E, Aksu FT, Çelik A, *et al*. Digit ratio (2D:4D) comparison between competitive age group swimmers and non-athletes, *Palestrica of the third millennium – Civilization and Sport*, 2017, **18**(4):206-209.

S64. Ceylan L, Küçük H, Ceylan T, *et al*. The 2nd:4th digit ratio and shooting skill performance in Basketball Players, *Mediterranean Journal of Sport Science*, 2022, **5**(3): 537-549.

S65. Azam Z, Zainuddin ZA, Kosni NA, *et al*. Influence of Digit Ratio (2D:4D) on Aerobic and Anaerobic Based Fitness Tests among Primary Schoolchildren, *Journal of Advances in Sports and Physical Education*, 2022, doi: [10.36348/jaspe.2022.v05i05.002](http://dx.doi.org/10.36348/jaspe.2022.v05i05.002)

S66. de laCruz-Sánchez E, García-Pallarés J, Torres-Bonete MD, *et al*. Can Our Fingers Alone Raise Us Up to the Sky? Analysis of the Digit Ratio Association with Success in Olympic Wrestling. *Coll Antropol*. 2015; **39**(3):515-9.

S67. Azam Z, Adanen NM, Adli M. Preliminary study: Digit ratio (2D:4D) Among U20 male and female rugby sevens state players, *Journal of Physical Education and Sport*, 2022, **22**(11):2763-2769.

S68. Tomaszewska A, Lubońska JA. 2D:4D digit ratio and its relationship to BMI, sporting choices and physiological predispositions among women, *Anthropological Review*, 2022, 85(2)**.**

S69. Akyol P, Tutkun E, Çebi M. The influence of hand finger length ratio on the motoric and functional dominance of women dancers athletes and sedentaries. *International Journal of Academic Research*, 2016, **8**(3).

S70. Lu H, Shen D, Wang L, *et al*. Digit ratio (2D:4D) and handgrip strength are correlated in women (but not in men) in Hui ethnicity. *Early Hum Dev*. 2017; **109**:21-25.

S71. Verma K, More V, Patel JK, *et al.* 2D:4D Ratio as a Predictor for Swimming Learning-A Pilot Study. *Journal of Clinical and Diagnostic Research*, 2021, 15(12):AC16-AC19

S72. Kogure GS, Silva RC, Miranda-Furtado CL *et al.* Hyperandrogenism enhances muscle strength after progressive resistance training, independent of body composition, in women with polycystic ovary syndrome. *J Strength Cond Res* 2018; **32**:2642–51.

S73. Konieczna A, Rachoń D, Owczarek K *et al.* Serum bisphenol A concentrations correlate with serum testosterone levels in women with polycystic ovary syndrome. *Reprod Toxicol* 2018; **82**:32–7.

S74. Deng Y, Zhang Y, Li S *et al.* Steroid hormone profiling in obese and nonobese women with polycystic ovary syndrome. *Sci Rep* 2017; **7**:14156.

S75. Couto Alves A, Valcarcel B, Mäkinen VP *et al.* Metabolic profiling of polycystic ovary syndrome reveals interactions with abdominal obesity. *Int J Obes (Lond)* 2017; **41**:1331–40.

S76. Han Y, Kim HS, Lee HJ *et al.* Metabolic effects of polycystic ovary syndrome in adolescents. *Ann Pediatr Endocrinol Metab* 2015; **20**:136–42.

S77. Keefe CC, Goldman MM, Zhang K *et al.* Simultaneous measurement of thirteen steroid hormones in women with polycystic ovary syndrome and control women using liquid chromatography-tandem mass spectrometry. *PLoS One* 2014; **9**:e93805.

S78. Barry JA, Parekh HS, Hardiman PJ. Visual-spatial cognition in women with polycystic ovarian syndrome: the role of androgens. *Hum Reprod* 2013; **28**:2832–7.

S79. Kandaraki E, Chatzigeorgiou A, Livadas S *et al.* Endocrine disruptors and polycystic ovary syndrome (PCOS): elevated serum levels of bisphenol A in women with PCOS. *J Clin Endocrinol Metab* 2011; **96**:E480–4.

S80. Economou F, Xyrafis X, Livadas S *et al.* In overweight/obese but not in normal-weight women, polycystic ovary syndrome is associated with elevated liver enzymes compared to controls. *Hormones (Athens)* 2009; **8**:199–206.

S81. Carmina E, Guastella E, Longo Ra *et al.* Correlates of increased lean muscle mass in women with polycystic ovary syndrome. *Eur J Endocrinol* 2009; **161**:583–9.

S82. Schattmann L, Sherwin BB. Testosterone levels and cognitive functioning in women with polycystic ovary syndrome and in healthy young women. *Horm Behav* 2007; **51**:587–96.

S83. Escobar-Morreale HF, Botella-Carretero JI, Villuendas G *et al.* Serum interleukin-18 concentrations are increased in the polycystic ovary syndrome: relationship to insulin resistance and to obesity. J Clin Endocrinol Metab 2004; **89**:806–11.

S84. Remsberg KE, Talbott EO, Zborowski JV *et al.* Evidence for competing effects of body mass, hyperinsulinemia, insulin resistance, and androgens on leptin levels among lean, overweight, and obese women with polycystic ovary syndrome. *Fertil Steril* 2002; **78**:479–86.

S85. Winters SJ, Talbott E, Guzick DS *et al.* Serum testosterone levels decrease in middle age in women with the polycystic ovary syndrome. *Fertil Steril* 2000; **73**:724–9.

S86. Conway GS, Agrawal R, Betteridge DJ *et al.* Risk factors for coronary artery disease in lean and obese women with the polycystic ovary syndrome. *Clin Endocrinol* 1992; **37**:119–25.

S87. Liu C, Liu K, Zhao X, *et al*. The Associations Between Alanine Aminotransferase and Other Biochemical Parameters in Lean PCOS. *Reprod Sci*. 2023; **30**(2):633-641.

S88. Keyif B, Goksever Celik H, Karamustafaoglu Balci B, *et al*. Serum betatrophin levels are significantly increased in obese patients compared to lean patients regardless to the presence of PCOS. *Gynecol Endocrinol*. 2020; **36**(8):678-681.

S89. Cundubey CR, Cam SD. Serum Phoenixin-14 levels of women with polycystic ovary syndrome increase proportionally with BMI. *Eur Rev Med Pharmacol Sci*. 2023; **27**(8):3519-3525.

S90. Sahin S, Eroglu M, Selcuk S, *et al*. Intrinsic factors rather than vitamin D deficiency are related to insulin resistance in lean women with polycystic ovary syndrome. *Eur Rev Med Pharmacol Sci*. 2014; **18**(19):2851-6.

S91. González F, Sia CL, Stanczyk FZ, *et al*. Hyperandrogenism exerts an anti-inflammatory effect in obese women with polycystic ovary syndrome. *Endocrine*. 2012; **42**(3):726-35.

S92. Grimmichová T, Vrbíková J, Matucha P, *et al*. Fasting insulin pulsatile secretion in lean women with polycystic ovary syndrome. *Physiol Res*. 2008; **57** Suppl 1:S91-S98.

S93. Mørch NF, Aziz M, Svendsen PF. Bone mass density in lean and overweight women with polycystic ovary syndrome. Scand J Clin Lab Invest 2022; **82**:210–17.

S94. Kazemi M, Jarrett BY, Parry SA *et* al. Osteosarcopenia in reproductive-aged women with polycystic ovary syndrome: a multicenter case-control study. J Clin Endocrinol Metab 2020; **105**:e3400–14.

S95. Pereira-Eshraghi CF, Chiuzan C, Zhang Y *et al*. Obesity and insulin resistance, not polycystic ovary syndrome, are independent predictors of bone mineral density in adolescents and young women. *Horm Res Paediatr* 2019; **92**:365–71.

S96. Ganie MA, Chakraborty S, Sehgal A *et al.* Bone mineral density is unaltered in women with polycystic ovary syndrome. *Horm Metab Res* 2018; **50**:754–60.

S97. McBreairty LE, Zello GA, Gordon JJ *et al.* Women with polycystic ovary syndrome have comparable hip bone geometry to age-matched control women. *J Clin Densitom* 2018; **21**:54–60.

S98. Karadağ C, Yoldemir T, Gogas Yavuz D. Determinants of low bone mineral density in premenopausal polycystic ovary syndrome patients. *Gynecol Endocrinol* 2017; **33**:234–7.

S99. Katulski K, Slawek S, Czyzyk A *et al*. Bone mineral density in women with polycystic ovary syndrome. *J Endocrinol Invest* 2014; **37**:1219–24.

S100. To WW, Wong MW. A comparison of bone mineral density in normal weight and obese adolescents with polycystic ovary syndrome. *J Pediatr Adolesc Gynecol* 2012; **25**:248–53.

S101. Mario FM, do Amarante F, Toscani MK, *et al*. Lean muscle mass in classic or ovulatory PCOS: association with central obesity and insulin resistance. *Exp Clin Endocrinol Diabetes* 2012; **120**:511–16.

S102. Schmidt J, Dahlgren E, Brännström M, *et al*. Body composition, bone mineral density and fractures in late postmenopausal women with polycystic ovary syndrome — a long-term follow-up study. *Clin Endocrinol (Oxf)* 2012; **77**:207–14.

S103. Kassanos D, Trakakis E, Baltas CS *et al*. Augmentation of cortical bone mineral density in women with polycystic ovary syndrome: a peripheral quantitative computed tomography (pQCT) study. *Hum Reprod* 2010; **25**:2107–14.

S104. Sum M, Warren M. Hypothalamic amenorrhea in young women with underlying polycystic ovary syndrome. *Fertil Steril* 2009; **92**:2106–8.

S105. Noyan V, Yucel A, Sagsoz N. The association of bone mineral density with insulin resistance in patients with polycystic ovary syndrome. *Eur J Obstet Gynecol Reprod Biol* 2004; **115**:200–5.

S106. Kirchengast S, Huber J. Body composition characteristics and body fat distribution in lean women with polycystic ovary syndrome. *Hum Reprod* 2001; **16**:1255–60.

S107. Yüksel O, Dökmetaş HS, Topcu S *et al*. Relationship between bone mineral density and insulin resistance in polycystic ovary syndrome. *J Bone Miner Metab* 2001; **19**:257–62.

S108. Good C, Tulchinsky M, Mauger D *et al.* Bone mineral density and body composition in lean women with polycystic ovary syndrome. *Fertil Steril* 1999; **72**:21–5.

S109. Adami S, Zamberlan N, Castello R, Tosi F, *et al*. Effect of hyperandrogenism and menstrual cycle abnormalities on bone mass and bone turnover in young women. *Clin Endocrinol (Oxf)*. 1998; **48**(2):169-73.

S110. Fawzy O, Elghaffar N, Mahmoud E, *et al*. Bone mineral density in relation to polycystic ovary syndrome: an insight into irisin and insulin. *The Scientific Journal of Al-Azhar Medical Faculty*, *Girls*, 2018, **2**:194-204

S111. Gao S, Cheng Y, Zhao L, *et al*. The relationships of irisin with bone mineral density and body composition in PCOS patients. *Diabetes Metab Res Rev*. 2016; **32**(4):421-8.

S112. Fighera TM, Dos Santos BR, Spritzer PM. Lean mass and associated factors in women with PCOS with different phenotypes. *PLoS One*. 2023, **5**;18(10):e0292623.

S113. Kalyan S, Patel MS, Kingwell E, *et al*. Competing Factors Link to Bone Health in Polycystic Ovary Syndrome: Chronic Low-Grade Inflammation Takes a Toll. *Sci Rep*. 2017,13;**7**(1):3432.

S114. Nunes E, Gallardo E, Morgado-Nunes S, *et al*. Steroid hormone levels and bone mineral density in women over 65 years of age. *Sci Rep* 2023; **13**:4925.

S115. Patalong-Wójcik M, Golara A, Sokołowska A *et al*. Associations of hormonal and metabolic parameters with bone mineralization in young adult females. *Nutrients* 2023; **15**:2482.

S116. Zhang H, Ma K, Li RM *et al*. Association between testosterone levels and bone mineral density in females aged 40–60 years from NHANES 2011–2016. *Sci Rep* 2022; **12**:16426.

S117. Al-Daghri NM, Yakout SM, Ansari MGA *et al*. Vitamin D metabolites and sex steroid indices in postmenopausal women with and without low bone mass. *Metabolites* 2021; **11**:86.

S118. Sasaki E, Chiba D *et al.* Reduced serum levels of anti-Mullerian hormone is a putative biomarker of early knee osteoarthritis in middle-aged females at menopausal transition. *Sci Rep* 2021; **11**:4931.

S119. Arpaci D, Saglam F, Cuhaci FN *et al*. Serum testosterone does not affect bone mineral density in postmenopausal women. *Arch Endocrinol Metab* 2015; **59**:292–6.

S120. Xu L, Wang Q, Wang Q *et al.* Concerted actions of insulin-like growth factor 1, testosterone, and estradiol on peripubertal bone growth: a 7-year longitudinal study. *J Bone Miner Res* 2011; **26**:2204–11.

S121. Bredella MA, Torriani M, Ghomi RH *et al*. Determinants of bone mineral density in obese premenopausal women. *Bone* 2011; **48**:748–54.

S122. Liu SZ, Tian LF, Xu P *et al.* Analysis of correlation between blood biochemical indicators and bone mineral density of post-menopausal women. *Mol Biol Rep* 2011; **38**:939–48.

S123. Rariy CM, Ratcliffe SJ, Weinstein R *et al*. Higher serum free testosterone concentration in older women is associated with greater bone mineral density, lean body mass, and total fat mass: the cardiovascular health study. *J Clin Endocrinol Metab* 2011; **96**:989–96.

S124. Maïmoun L, Coste O, Jaussent A *et al.* Bone mass acquisition in female rhythmic gymnasts during puberty: no direct role for leptin. *Clin Endocrinol* 2010; **72**:604–11.

S125. Yong M, Atkinson C, Newton KM *et al*. Associations between endogenous sex hormone levels and mammographic and bone densities in premenopausal women. *Cancer Causes Control* 2009; **20**:1039–53.

S126. Sowers MR, Jannausch M, McConnell D *et al*. Hormone predictors of bone mineral density changes during the menopausal transition. *J Clin Endocrinol Metab* 2006; **91**:1261–7.

S127. Bagur A, Oliveri B, Mautalen C *et al.* Low levels of endogenous estradiol protect bone mineral density in young postmenopausal women. *Climacteric* 2004; **7**:181–8.

S128. Bonofiglio D, Garofalo C, Catalano S *et al*. Low calcium intake is associated with decreased adrenal androgens and reduced bone age in premenarcheal girls in the last pubertal stages. *J Bone Miner Metab* 2004; **22**:64–70.

S129. García-Pérez MA, Moreno-Mercer J, Tarín JJ *et al*. Relationship between PTH, sex steroid and bone turnover marker measurements and bone density in recently postmenopausal women. *Maturitas* 2003; **45**:67–74.

S130. Gravholt CH, Lauridsen AL, Brixen K *et al*. Marked disproportionality in bone size and mineral, and distinct abnormalities in bone markers and calcitropic hormones in adult turner syndrome: a cross-sectional study. *J Clin Endocrinol Metab* 2002; **87**:2798–808.

S131. Buist DS, LaCroix AZ, Barlow WE *et al*. Bone mineral density and endogenous hormones and risk of breast cancer in postmenopausal women (United States). *Cancer Causes Control* 2001; **12**:213–22.

S132. Zofková I, Bahbouh R, Hill M. The pathophysiological implications of circulating androgens on bone mineral density in a normal female population. *Steroids* 2000; **65**:857–61.

S133. Orozco P, Navarro MA, Nolla JM. Salivary testosterone is associated with higher lumbar bone mass in premenopausal healthy women with normal levels of serum testosterone. *Eur J Epidemiol* 2000; **16**:907–12.

S134. Greendale GA, Edelstein S, Barrett-Connor E. Endogenous sex steroids and bone mineral density in older women and men: the Rancho Bernardo Study. *J Bone Miner Res* 1997; **12**:1833–43.

S135. Perry HM 3rd, Horowitz M, Morley JE *et al*. Aging and bone metabolism in African American and Caucasian women. *J Clin Endocrinol Metab* 1996; **81**:1108–17.

S136. Murphy S, Khaw KT, Sneyd MJ *et al*. Endogenous sex hormones and bone mineral density among community-based postmenopausal women. *Postgrad Med J* 1992; **68**:908–13.

S137. Daniel M, Martin AD, Drinkwater DT. Cigarette smoking, steroid hormones, and bone mineral density in young women. *Calcif Tissue Int* 1992; **50**:300–5.

S138. Shiraki M, Ito H, Fujimaki H *et al*. Relation between body size and bone mineral density with special reference to sex hormones and calcium regulating hormones in elderly females. *Endocrinol Jpn* 1991; **38**:343–9.

S139. Simberg N, Tiitinen A, Silfvast A, *et al*. High bone density in hyperandrogenic women: effect of gonadotropin-releasing hormone agonist alone or in conjunction with estrogen-progestin replacement. *J Clin Endocrinol Metab*. 1996; **81**(2):646-51.

S140. Glintborg D, Andersen M, Hagen C, *et al*. Higher bone mineral density in Caucasian, hirsute patients of reproductive age. Positive correlation of testosterone levels with bone mineral density in hirsutism. *Clin Endocrinol (Oxf)*. 2005; **62**(6):683-91.

S141. Lambrinoudaki I, Christodoulakos G, Aravantinos L, *et al*. Endogenous sex steroids and bone mineral density in healthy Greek postmenopausal women. *J Bone Miner Metab*. 2006; **24**(1):65-71.

S142. Yilmaz D, Ersoy B, Bilgin E, *et al*. Bone mineral density in girls and boys at different pubertal stages: relation with gonadal steroids, bone formation markers, and growth parameters. *J Bone Miner Metab*. 2005; **23**(6):476-82.

S143. Wang Q, Nicholson PH, Suuriniemi M, *et al*. Relationship of sex hormones to bone geometric properties and mineral density in early pubertal girls. *J Clin Endocrinol Metab*. 2004; **89**(4):1698-703

S144. Smoliga JM, Fogaca LK, Siplon JS et al. Giving science the finger—is the second-to-fourth digit ratio (2D:4D) a biomarker of good luck? A cross sectional study. *BMJ* 2021; **375**:e067849.

S145. Arazi H, Eghbali E. 25-Hydroxyvitamin D levels and its relation to muscle strength, maximal oxygen consumption, and body mass index in young and middle adulthood women. *Int J Womens Health* 2019; **11**:57–64.

# S146. Arazi H, Eghbali E, Saeedi T *et al.* The relationship of physical activity and anthropometric and physiological characteristics to bone mineral density in postmenopausal women. *J Clin Densitometry* 2016; 19:382–8.

S147. Buyuk GN, Ozdemir EU, Halilzade I *et al*. Anogenital index and bone mineral density associations after natural and surgical menopause: a preliminary study. *Rev Assoc Med Bras* 2022; **68**:1737–41.

S148. Fawzy T, Muttappallymyalil J, Sreedharan J *et al*. Association between body mass index and bone mineral density in patients referred for dual-energy X-ray absorptiometry scan in Ajman, UAE. *J Osteoporos* 2011; **2011**:876309.

S149. Song J, Zhang R, Lv L *et al.* The relationship between body mass index and bone mineral density: a Mendelian randomization study. *Calcif Tissue Int* 2020; **107**:440–5.
